# Neuroimaging in inborn errors of immunity: More than infections

**DOI:** 10.1007/s00234-025-03895-5

**Published:** 2026-01-26

**Authors:** Pedro Sousa Brandão, Carla Conceição, João Farela Neves

**Affiliations:** 1 Department of Neuroradiology, Unidade Local de Saúde São José, Lisbon, Portugal; 2 Centro Clínico Académico de Lisboa, Lisbon, Portugal; 3 Primary Immunodeficiencies Unit, Hospital Dona Estefânia, Unidade Local de Saúde São José, Lisbon, Portugal; 4 Chronic Diseases Research Center (CEDOC), Nova Medical School, Lisbon, Portugal

**Keywords:** Inborn errors of immunity, Immunologic deficiency syndromes, Neuroimaging, Magnetic resonance imaging

## Abstract

**Purpose:**

Inborn errors of immunity (IEIs) are a heterogeneous group of germline disorders whose recognition is increasing but which remain underdiagnosed and associated with significant pediatric morbidity and mortality. This review aims to systematize the main neuroimaging manifestations of IEIs, addressing how neuroradiologists can identify characteristic patterns to support earlier diagnosis and guide clinical management.

**Methods:**

A narrative review of IEIs with neurological involvement was performed, focusing on reported primary and secondary neuroimaging features across major IEI categories. Imaging findings were correlated with underlying immune defects, typical clinical phenotypes, and their impact on diagnostic workup, risk assessment, and genetic counseling.

**Results:**

IEIs demonstrate a broad neurological spectrum that extends beyond recurrent infections to encompass autoinflammatory, autoimmune, allergic, and malignant phenotypes with central nervous system involvement. Neuroimaging reveals both primary manifestations directly related to immune dysregulation and secondary features, allowing recognition of patterns in IEI subgroups that can provide crucial diagnostic clues in otherwise non-specific clinical scenarios.

**Conclusion:**

Neurological involvement in IEIs is not uncommon and may be radiologically detectable, making imaging an important ancillary tool in their evaluation. Systematic recognition of primary and secondary neuroimaging features by neuroradiologists can raise suspicion for an underlying IEI, prompting appropriate immunologic and genetic work-up. Earlier diagnosis is crucial to enable timely implementation of preventive or targeted therapies, with the potential to improve long-term outcomes.

## Introduction

Human inborn errors of immunity (IEIs), formerly known as primary immunodeficiencies, are a heterogeneous group of inherited disorders caused by specific germline variants affecting immune system development and/or function [1–4].

Most IEIs are individually rare, but epidemiologic studies suggest that they are more common than previously thought [5] and their incidence rate as a group was 10.3 per 100,000 person-years from 2001 to 2006 according to the United States population-based cohort [3]. The global incidence of IEIs has been increasing during the past decades mostly due to improvements in diagnosis and genetic characterization, but their recognition remains limited [3, 6] and they still represent a major cause of morbidity and mortality, particularly in the pediatric population [4]. To promote further understanding of the pathogenic mechanisms and for educational, clinical, and research purposes, groups dedicated to the study of IEIs have been established, including the Expert Committee of the International Union of Immunological Societies (IUIS). As reported by the most recent 2022 Update on the Classification from the IUIS Expert Committee, there are currently 485 genetic defects identified as causing IEIs [1, 4].

IEIs are classically classified based on the primary immune component involved as deficiencies of innate immune response (including phagocytes, complement, and TLR-mediated signaling and the IFN-gamma/IL-12 axis disorders), adaptive immune response (encompassing B cells and antibodies, T cells and combined immunodeficiencies), and other types (including more complex phenotypes, such as immunodeficiencies associated with other major defects, autoimmune, autoinflammatory, and immunodeficiency syndromes) [2, 3]. Conversely, the 2022 IUIS classification categorizes IEIs into ten main groups based on common pathogenesis and clinical phenotype to assist the physician in identifying the most probable diagnosis [1, 4].

Patients often present with increased susceptibility to infections, characterized by recurrent, severe, and difficult-to-treat episodes. Nonetheless, these disorders can exhibit a wide range of clinical presentations, and immune dysregulation has been increasingly recognized as either the main phenotype or a component of several IEIs, manifesting as inflammation, autoimmunity, allergy, lymphoproliferation, or malignancies [1–3].

Given the growing diversity of IEIs phenotypes, diagnosis is often challenging, with up to 60% of patients remaining undiagnosed until adulthood [2, 7, 8]. Early diagnosis requires a high degree of clinical suspicion where imaging can play an important ancillary role [8, 9]. In most cases, timely diagnosis significantly improves prognosis by allowing for the early implementation of preventive or specific therapeutic measures [3]. A definitive diagnosis can be achieved in approximately 75% of cases and is indispensable for accurate genetic risk assessment and counseling.

Neurologic involvement in IEIs is not uncommon, but the literature primarily addresses clinical aspects [7, 10–16]. In this article, we provide a systematized overview of the main neuroimaging findings in IEIs, emphasizing the importance of neuroradiologists’ awareness of these features.

## Neurologic manifestations

Neurologic manifestations in patients with IEIs have been reported more frequently in those with combined immunodeficiencies, followed by antibody immunodeficiencies and phagocyte defects [13]. These may be the initial presentation in some IEIs, representing an important cause of morbimortality in affected patients [7, 12]. The estimated prevalence of neurologic involvement in IEIs varies from 1.8% to 2.5%, but its exact frequency is not known [12, 13].

Different mechanisms have been implicated in the neuropathogenesis including impaired enzymatic function with accumulation of cytotoxic products, defective nucleic acids break repair, abnormal intracellular trafficking of synaptic vesicles, increased neuronal apoptosis, lymphohistiocytic infiltration, neurovascular abnormalities, and autoinflammatory processes [7].

There is a wide spectrum of neurologic manifestations reported in IEIs that include microcephaly, seizures/epilepsy, cognitive deficit, global developmental delay, ataxia, encephalopathy, hypotonia, motor dysfunction, behavioral disturbances, predisposition to stroke, central or peripheral neuropathy, chronic aseptic meningitis, and abnormal eye movements [7, 12, 13].

Based on the etiopathogenesis, these can be classified as either primary, when they represent core features of the disease or direct consequences of the underlying genetic defect, or secondary, resulting from infections, lymphoproliferative or malignant processes, or other forms of immune dysregulation such as autoimmunity and hyperinflammation [7, 11–14].

## Neuroimaging findings

According to the largest case study in the literature, 74% of patients with IEIs and neurologic involvement display abnormalities on brain magnetic resonance imaging (MRI) [12], including cerebellar or cerebral atrophy, leukoencephalopathy, gray matter lesions, hydrocephalus, posterior reversible encephalopathy syndrome (PRES), intracranial hemorrhage, vascular anomalies, central nervous system (CNS) congenital malformations, and vasculitis [7, 12, 13].

Recognizing the potential neurologic presentations of some IEIs and their corresponding imaging findings is essential for both physicians and neuroradiologists. Familiarity with these manifestations is crucial for early diagnosis and facilitates appropriate treatment and genetic counseling, ultimately aiming prevent or mitigate neurologic sequelae. Imaging plays a pivotal role not only in diagnosing primary and secondary neurologic features but also in guiding biopsies and monitoring disease progression.

Given the broad range phenotypes, no published works offer a comprehensive and systematic approach to the neuroimaging features of IEIs. In this regard, we outline the most relevant imaging findings of the IEIs with neurologic involvement (summarized in Table 1), categorizing them into primary and secondary (infectious or non-infectious – inflammatory, neurodegenerative, or malignant). However, classification into these etiopathogenic groups is not always feasible [13].

**Table 1:** Main neuroimaging findings in inborn errors of immunity

Primary features	
Microcephaly	NBS; MKD; Cernunnos/XLF deficiency; DNA ligase IV deficiency; DNA PKcs deficiency; RIDDLE syndrome; Roifman syndrome; LAD2, Vici syndrome; ICF syndrome; Cohen syndrome; Mulvihill-Smith syndrome; Rubinstein-Taybi syndrome; Dubowitz syndrome; MYSM1 deficiency; ERCC6L2 disease; TGFB1 deficiency; 3-methylglutaconic aciduria
Facial dysmorphism	NBS; DiGeorge syndrome; Bloom syndrome; DNA ligase IV deficiency; RIDDLE syndrome; Roifman syndrome; ICF syndrome; HPS type 2; Kabuki syndrome; NOMID/CINCA syndrome; congenital disorders of glycosylation
Vertebral abnormalities	SPENCDI; Roifman syndrome; SIOD; EXTL3 deficiency; cartilage-hair hypoplasia; ADA deficiency (“bone-in-bone” appearance of the vertebral bodies)
Secondary features	
CNS infection	
Viral	XLA; TLR3 pathway defects; DBR1 deficiency; RANBP2 gene mutations (ANE); NEMO deficiency; STAT1 deficiency; CVID; DOCK8 deficiency; GATA2 deficiency; SNORA31 deficiency; ATG4A deficiency; MAP1LC3B2 deficiency; CVID (PML)
Bacterial	CGD; IL12-gamma interferon and TLR pathways defects (for example, IRAK4 and MYD88 deficiencies)
Fungal	CGD; CARD9 deficiency
Inflammation	
Hemophagocytic lymphohistiocytosis	XLP1 and 2; FHL syndromes without hypopigmentation; CHS; GS2; HPS2 and 10; CEBPE neofunction; CD27 deficiency; CDC42 deficiency; TIM3 deficiency
CNS vasculitis	AR-HIES DOCK8 deficiency; XLP1 and 2; Blau syndrome; CMC; PNP deficiency; CVID
Neurodegeneration	AT; AGS; HHS; CRMCC; CHS
Malignancy	WAS; XLP1 and 2; AT; NBS (in particular medulloblastoma); Bloom syndrome; DNA ligase IV deficiency; DCLRE1C deficiency; DNA PKcs deficiency; Cernunnos/XLF deficiency; RIDDLE syndrome; AR-HIES DOCK8 deficiency; ADA deficiency
Other features	
Intracranial hemorrhage	AR-HIES DOCK8 deficiency; WAS; HLH (particularly in CHS); DADA2
Predisposition to stroke	AR-HIES DOCK8 deficiency; DADA2; SIOD; PNP deficiency; Blau syndrome; LAD2, NOMID/CINCA syndrome; AGS type 5 (SAMHD1 gene); SDS
Cerebrovascular anomalies	AD-HIES STAT3 deficiency; DADA2; AGS type 5
Ocular and optic nerve anomalies	Bloom syndrome; Roifman syndrome; IP; NOMID/CINCA syndrome; CMRCC
Parenchymal calcifications	AGS; HHS; CRMCC; SPENCDI; CANDLE syndrome; ISG15 deficiency

### Primary features

Nijmegen breakage syndrome (NBS) is a classic example of an IEI with prominent primary CNS involvement. It is an autosomal recessive syndrome caused by mutations in the NBS1 or nibrin gene, resulting in abnormal DNA repair [1]. Patients exhibit microcephaly, growth retardation, dysmorphic facial features (bird-like appearance), combined immunodeficiency, increased radiation sensitivity, and chromosomal instability [7]. Severe microcephaly is observed in all patients, usually at birth, though it can occasionally develop within the first months of life [7, 11, 12].

Typical neuroimaging findings in NBS include microcephaly, with reduced volume of the frontal lobes and narrow frontal horns of the lateral ventricles, accompanied by a simplified gyral pattern [7, 12, 17]. Partial agenesis of the corpus callosum, limbic system maldevelopment anomalies, and abnormal cerebrospinal fluid (CSF) spaces communicating with the ventricular system have also been reported [17].

Mevalonate kinase deficiency (MKD) is an autosomal recessive disorder affecting cholesterol biosynthesis. It is characterized by periodic fever and leukocytosis with high IgD levels, but cholesterol levels are typically normal, and the pathogenesis remains unclear [1]. Various CNS abnormalities may be present, such as microcephaly, dolicocephaly, and wide irregular fontanels. In milder cases, ataxia after preschool age is the major neurologic feature, attributable to progressive cerebellar atrophy, which can also be depicted on neuroimaging studies [7].

**Fig. 1:**
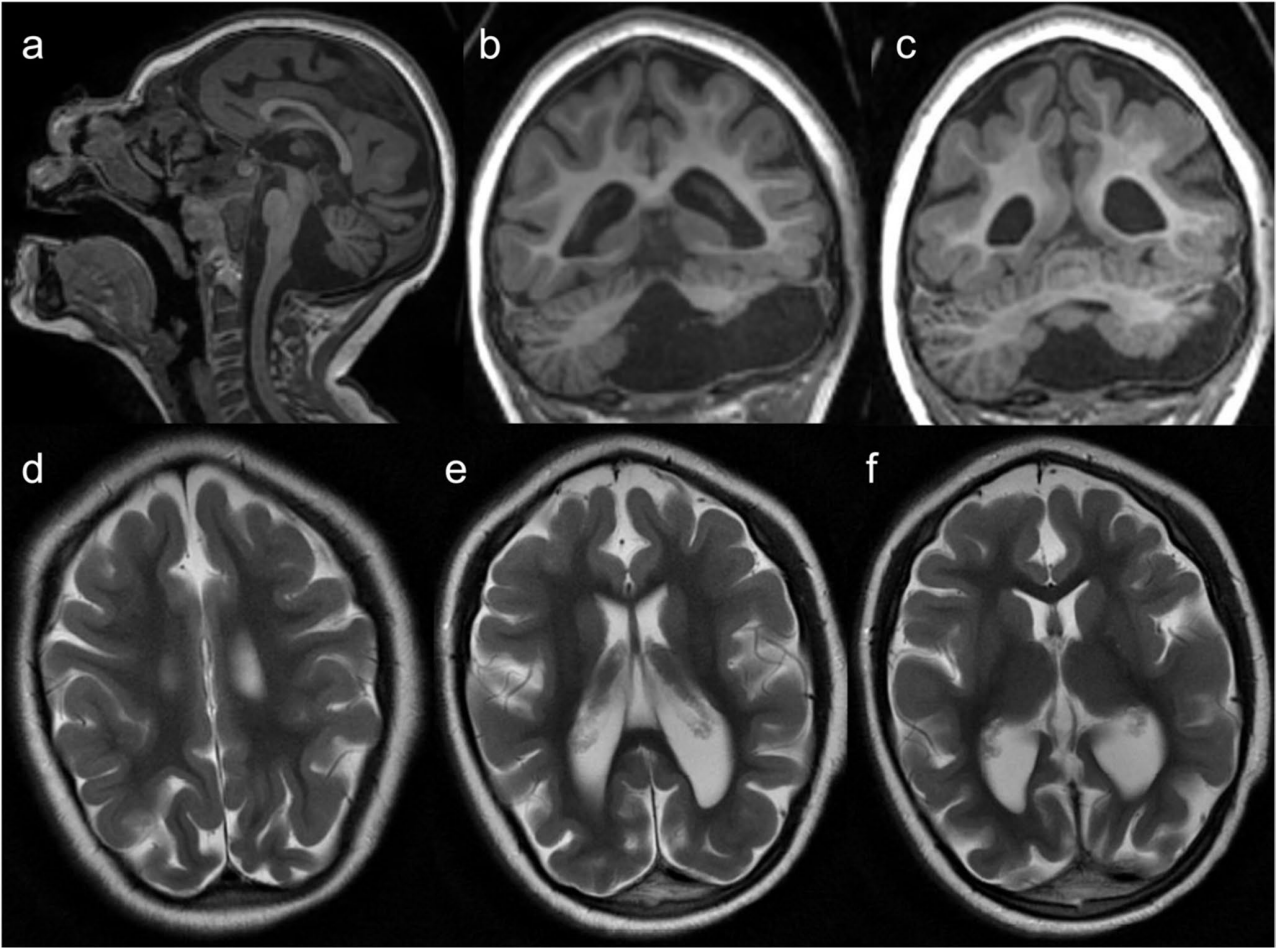
Immunodeficiency, centromeric region instability and facial anomalies syndrome (ICF) type 2. A 5-year-old male patient with a history of epilepsy, cerebral palsy, microcephaly, and facial anomalies (micrognathia, macroglossia, low-set ears, hypertelorism, and epicanthal folds) presenting with EBV-associated polymorphic T-cell lymphoproliferative disease. Next-generation sequencing panel of EBV-susceptibility related genes identified a homozygous ZBTB24 mutation. Brain MRI (**a** – sagittal T1, **b** and **c** – coronal T1, and **d** to **f** – axial T2) demonstrates microcephaly with a simplified gyral pattern, upward displacement of the hypoplastic left cerebellar hemisphere, and asymmetric dilation of the fourth ventricle, connected to an ipsilateral retrocerebellar cyst. These findings resemble the ‘tilted telephone receiver’ sign, characteristic of PHACES syndrome

Other IEIs presenting with microcephaly include Cernunnos/XLF deficiency, DNA ligase IV deficiency, DNA PKcs deficiency, RNF168 deficiency/radiosensitivity, immune deficiency, dysmorphic features, and learning difficulties (RIDDLE) syndrome, MOPD1 deficiency (Roifman syndrome), leukocyte adhesion deficiency type 2 (LAD2), EPG5 deficiency (Vici syndrome), immunodeficiency, centromeric region instability and facial anomalies (ICF) syndrome (Fig. 1), Cohen syndrome, Mulvihill-Smith syndrome, Rubinstein-Taybi syndrome, Dubowitz syndrome, MYSM1 deficiency, ERCC6L2 deficiency, TGFB1 deficiency, 3-methylglutaconic aciduria, and dyskeratosis congenita (DKC) such as Hoyeraal-Hreidarsson syndrome [1, 3, 7, 14, 16]. Furthermore, a dysmorphic facies is characteristic of several IEIs, including DiGeorge syndrome, Bloom syndrome, DNA ligase IV deficiency, RIDDLE syndrome, Roifman syndrome, ICF syndrome, Hermansky-Pudlak syndrome (HPS) type 2, Kabuki syndrome, neonatal onset multisystem inflammatory disorder, and congenital disorders of glycosylation [1, 3, 7]. Neural tube defects can also occur in DiGeorge syndrome [10].

Incontinentia pigmenti (IP), also known as Bloch–Sulzberger syndrome, is a rare X-linked dominant genodermatosis, usually lethal in utero in males. It is caused by mutations in the IKBKG (inhibitor of kappa B kinase gamma, previously NEMO) gene, which encodes a modulator of the nuclear factor kappa B (NF-κB) signaling pathway [18, 19].

Although patients with IP do not exhibit consistent immunological abnormalities, other pathogenic variants of the IKBKG gene are known to cause anhidrotic ectodermal dysplasia with immunodeficiency, as well as OLEDAID syndrome (osteopetrosis, lymphedema, anhidrotic ectodermal dysplasia, and immune deficiency) [1, 3].

One the major diagnostic criteria for IP, updated by Minić et al.. in 2014, is the presence of distinctive skin lesions distributed along Blashcko’s lines. These lesions progress through four often overlapping stages: neonatal bullous rash (vesiculobullous), verrucous plaques (verrucous), hyperpigmented swirling patterns (hyperpigmented), and finally the atrophic/hypopigmented stage [18, 20, 21].

Noncutaneous anomalies are present in up to 80% of patients and include ectodermal dysplasia (peg-shaped teeth, hypodontia and hypohidrosis), ophthalmologic disorders (most commonly a retrolental mass with dysplastic retinal detachment), and CNS abnormalities [18, 20].

Neurologic disease has an overall case prevalence of 30%, typically presenting as a destructive encephalopathy of neonatal or infantile onset without evidence of CNS infection. It commonly develops within the first year of life and constitutes one of the major causes of death [18, 19, 22].

**Fig. 2:**
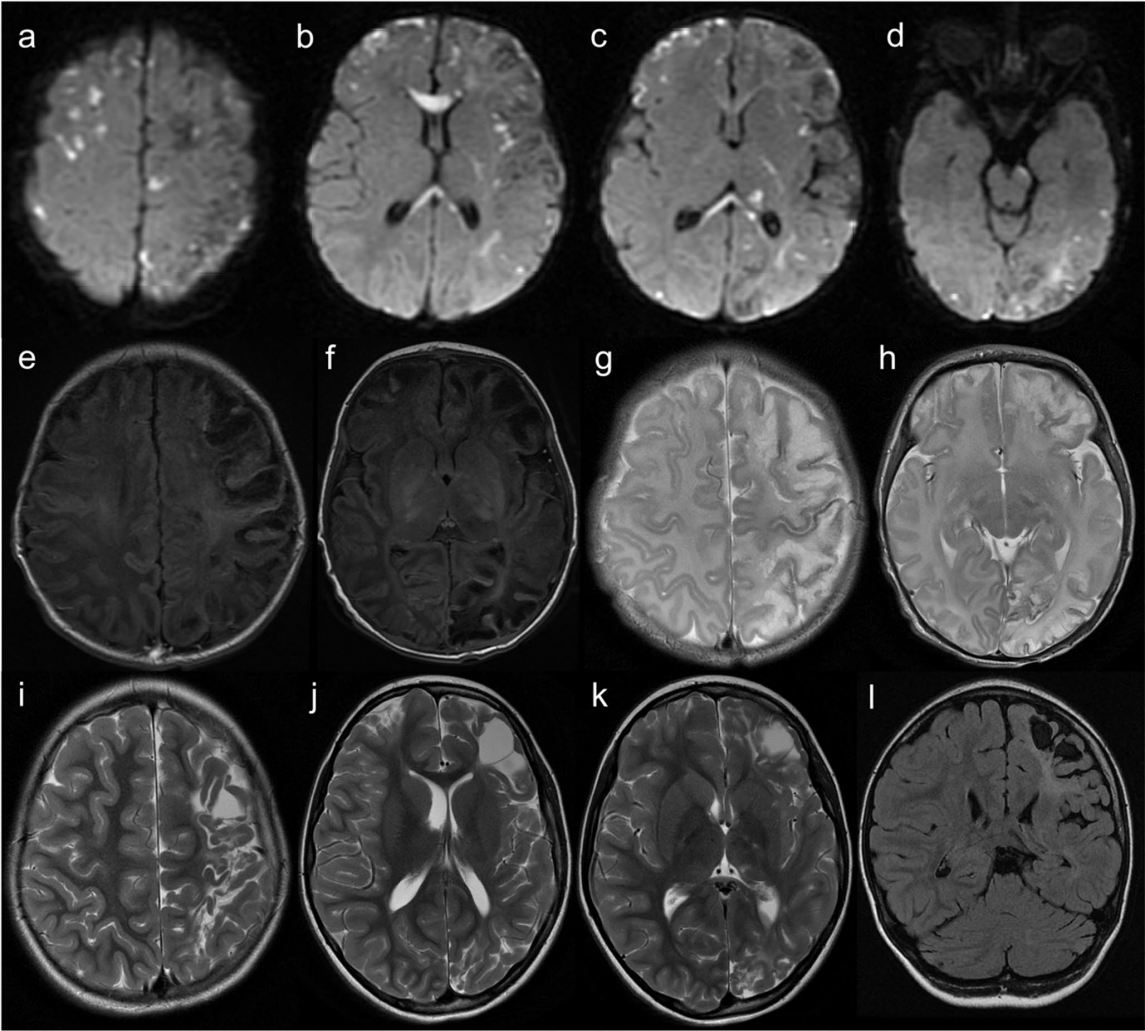
Incontinentia pigmenti. A 12-day-old female patient with seizures and a vesiculobullous skin rash. (**a** to **d**) Axial b1000 DWI demonstrates multiple hyperintense cortical and subcortical lesions (corresponding low signal on ADC maps is not shown) in the cerebral hemispheres, corpus callosum, left thalamus, and ipsilateral posterior limb of the internal capsule and cerebral peduncle. (**e** and **f**) Axial T2 and (**g** and **h**) FLAIR images reveal extensive subcortical white matter hyperintensities, predominantly in the left cerebral hemisphere. (**i**,** j**, and **k** – axial T2; **l** – coronal FLAIR) Follow-up brain MRI after 7 years shows progression to left cerebral hemiatrophy and white matter cavitation

As depicted in Fig. 2, brain MRI shows subcortical and periventricular white matter disease, cortical lesions, hemorrhagic changes, hypoplasia of the corpus callosum myelination delay, polymicrogyria, and other malformations of cortical development. Multifocal punctate or patchy areas of DWI (diffusion-weighted imaging) hyperintensity with corresponding low signal on ADC (apparent diffusion coefficient) maps are common in the deep and/or subcortical white matter during the acute phase, but can also be observed in the basal ganglia, thalami, cerebral cortex, corpus callosum, cerebellum, and cerebral peduncles [19, 22]. Although ischemia due to impaired cerebral perfusion is a potential mechanism, the more likely contributing factor is vascular dysfunction related to the disruption of the NF-κB signalling pathway, leading to blood-brain barrier disruption and neuroinflammation [19, 23, 24]. Progression to white matter cavitation and parenchymal atrophy is frequent on follow-up imaging [22].

Approximately 20 to 40% of IP patients have associated ocular abnormalities, including retinopathy, congenital cataracts and microphthalmia [22].

Hyper-IgE syndromes (HIES) are a complex group of distinct diseases characterized by elevated serum IgE levels, often accompanied by eosinophilia and an increased susceptibility to infections [1, 7, 10, 25]. Severe atopic eczema and multiple allergies are typical [14].

Autosomal dominant HIES due to STAT3 deficiency (Job syndrome) is associated with characteristic skeletal, connective tissue, and vascular abnormalities, such as coronary and cerebral aneurysms, Chiari I malformation and focal parenchymal hyperintensities on T2-weighted images. Neurologic complications in autosomal recessive HIES due to DOCK8 deficiency include CNS vasculitis, ischemic stroke, and subarachnoid hemorrhage [1, 7, 10, 25].

**Fig. 3:**
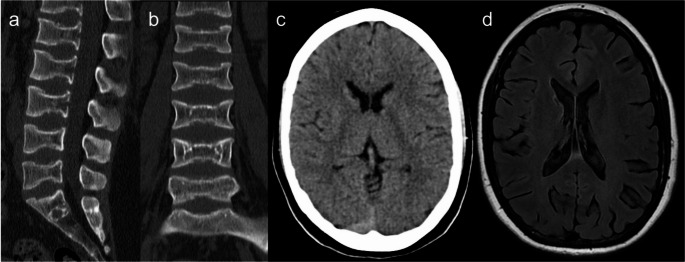
Spondyloenchondrodysplasia with immune dysregulation (SPENCDI). A 19-year-old female patient with short stature, paraparesis, and systemic lupus erythematosus. (**a** and **b**) Lumbar spine CT scan shows platyspondyly. (**c**) No intracranial calcifications are observed on head CT scan. (**d**) FLAIR images reveal a small cavitated lesion in the head of the right caudate nucleus with surrounding gliosis, likely of unrelated vascular etiology

Spondyloenchondrodysplasia with immune dysregulation (SPENCDI) is an autosomal recessive autoinflammatory disorder caused by mutations in the ACP5 gene, which encodes tartrate-resistant acid phosphatase [1, 7, 26], leading to an altered state of osteopontine phosphorylation with dysregulation of bone, immune and neurological systems [14, 27]. Patients present with spondylometaphyseal dysplasia characterized by disproportionate short stature due to platyspondyly (Fig. 3), and a wide spectrum of autoimmune phenotypes due to upregulation of type I interferon signaling, including systemic lupus erythematosus-like autoimmunity, hemolytic anemia and thrombocytopenia [1, 4, 14, 27]. Neurologic involvement is frequent and includes progressive spastic tetraparesis and intracranial calcifications, present in about half of the cases [1, 7, 14], typically symmetrical in the globus pallidus, putamen, subcortical white matter, or deep cortical gray matter [26, 27].

Schimke immunoosseous dysplasia (SIOD) and Roifman syndrome are characterized by skeletal spondyloepiphyseal dysplasia, which presents as ovoid, mildly flattened vertebral bodies in SIOD, and irregular, wavy vertebral plates in Roifman syndrome, both leading to disproportionate short stature [1, 3, 7]. Short stature is also a key feature in many IEIs, including cartilage-hair hypoplasia and immunoskeletal dysplasia with neurodevelopmental abnormalities (EXTL3 deficiency) [1, 3].

### Secondary features

#### Infectious

**Fig. 4:**
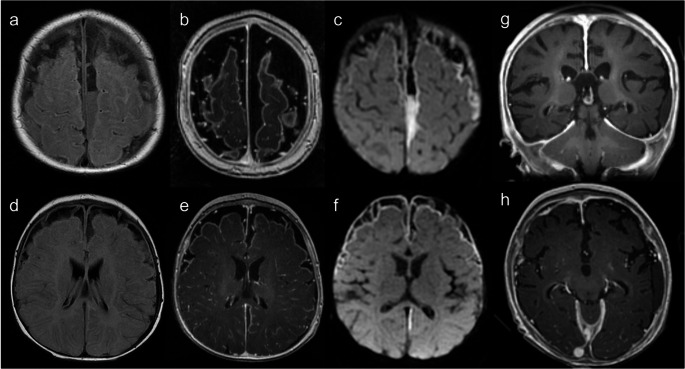
Immunodeficiency under investigation. A 3-month-old female patient with *Salmonella* meningitis. The patient has a history of neonatal sepsis without an identified causative agent and *Campylobacter jejuni* bacteremia in the context of acute gastroenteritis. Brain MRI (**a** and **d** – axial FLAIR, **b** and **e** – gadolinium-enhanced axial T1, and **c** and **f** – axial b1000 DWI) demonstrates extensive leptomeningeal involvement, with a fluid level of purulent material in the left parafalcine region, but no hydrocephalus or visible parenchymal abscesses. (g and h) Gadolinium-enhanced axial T1-weighted images 2 months after treatment show a favorable evolution of the leptomeningeal infection; however, there is generalized supra- and infratentorial pachymeningeal thickening and mild diffuse cerebral atrophy

**Fig. 5:**
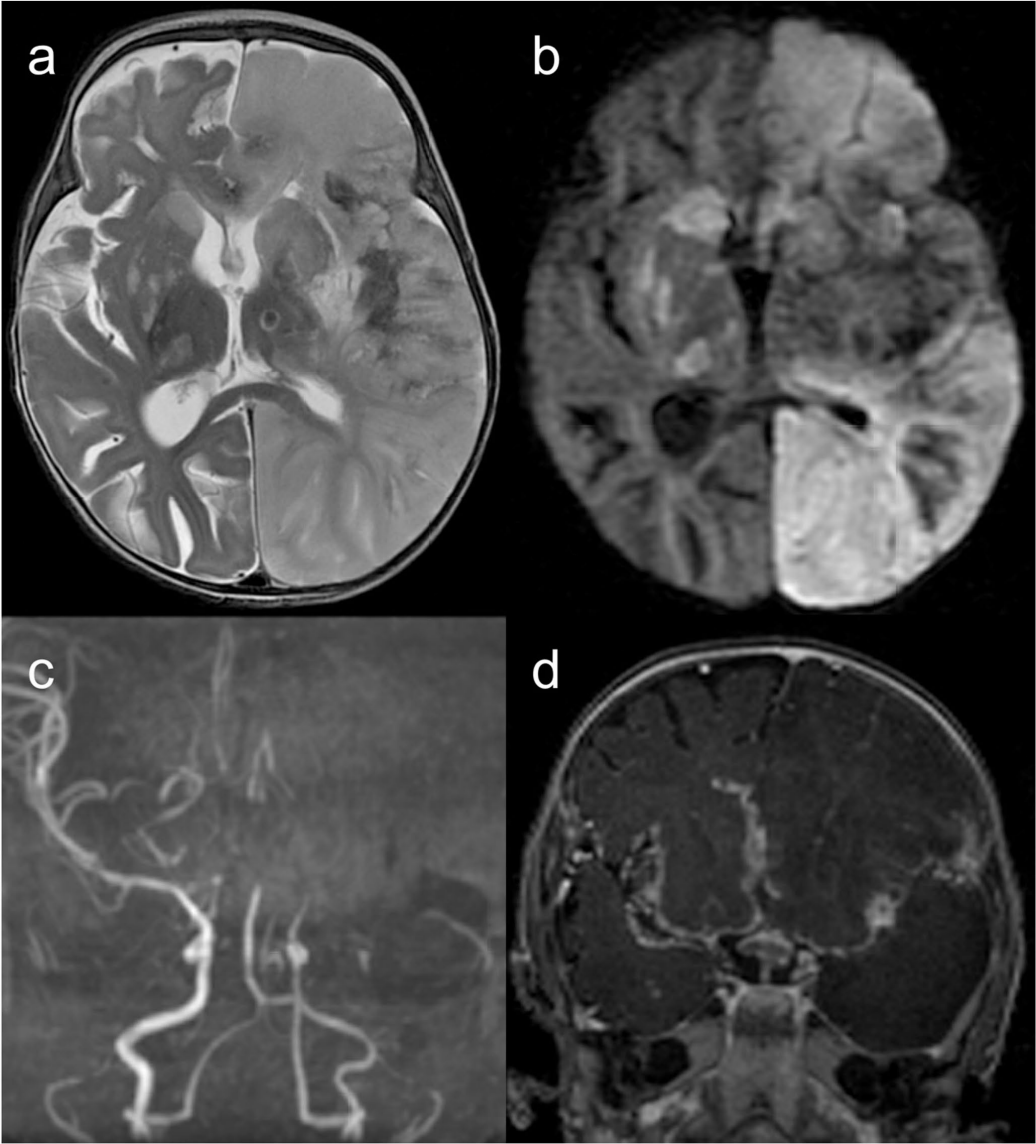
Immunodeficiency under investigation. A 6-month-old female patient with tuberculous meningitis complicated by ischemic stroke. Brain MRI (**a** – axial T2, **b **– axial b1000 DWI, **c** – coronal maximum intensity projection 3D TOF MR angiography, and** d** – gadolinium-enhanced coronal T1) reveals basal-predominant leptomeningeal enhancement and an acute infarction in the left cerebral hemisphere due to occlusion of the ipsilateral supraclinoid internal carotid artery. Additionally, there is hydrocephalus and bilateral acute ischemic lesions in the basal ganglia and thalami, attributed to small vessel vasculitis

**Fig. 6:**
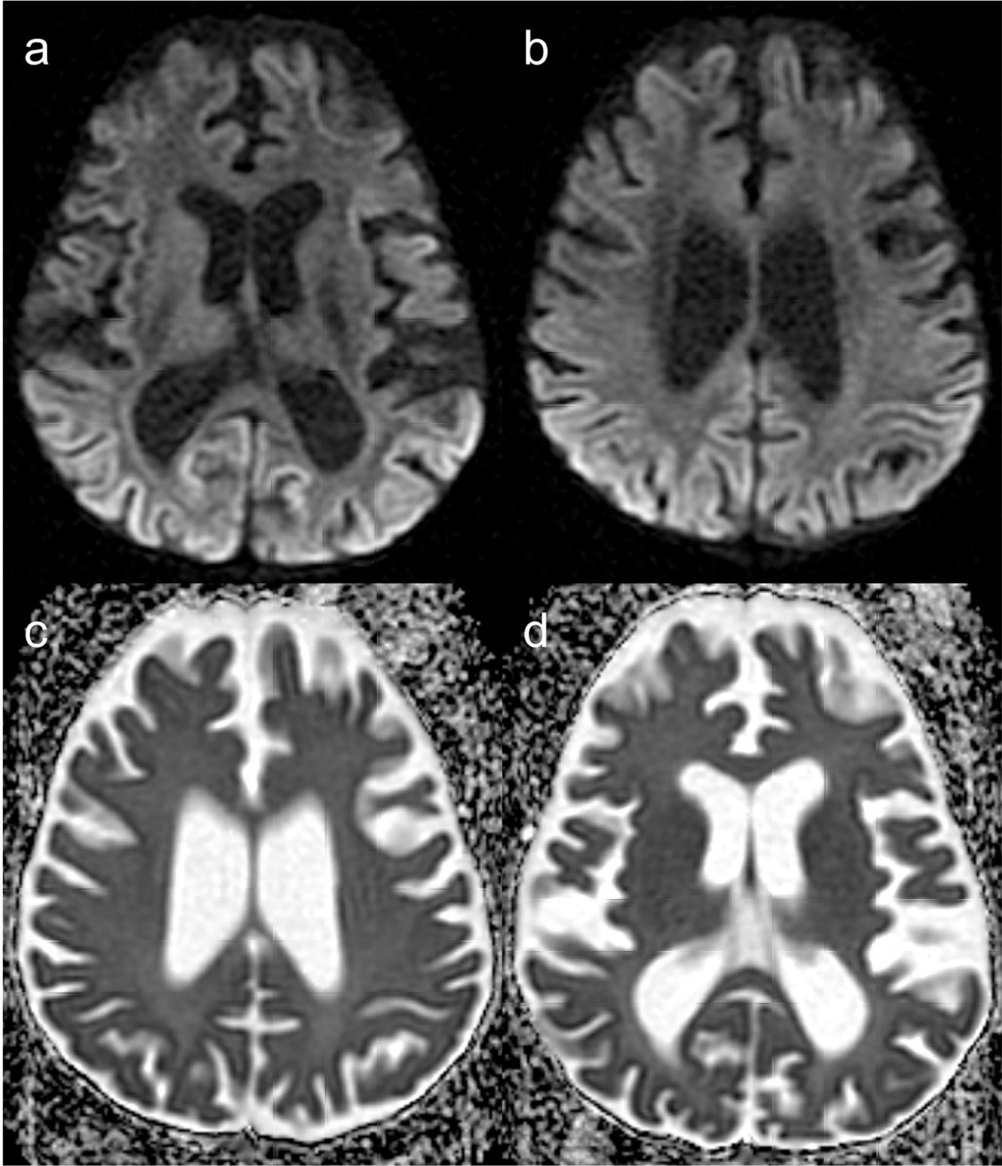
Severe combined immunodeficiency with no identified gene. A 2-year-old female patient with systemic JCV infection undergoing cidofovir treatment. Diffusion MR imaging (**a** and **b **– b1000, and **c** and **d** – ADC maps) depict extensive cortical areas of DWI hyperintensity and corresponding low signal on ADC maps, predominantly in the parieto-occipital regions. These findings have been sporadically reported in cases of encephalopathy secondary to lytic JCV infection of cortical pyramidal neurons. Diffuse cerebral atrophy is also present, likely related to prolonged immunosuppression for severe graft-versus-host disease following hematopoietic stem cell transplantation

Depending on the underlying genetic defect, clinical presentation of IEIs may be dominated by increased susceptibility to a selective or broad spectrum of infectious pathogens [2, 3] (Figs. 4, 5 and 6). The primary immune component involved justifies the relative phenotypical homogeneity between the classically defined immunodeficiency types.

CNS infections are associated with a relatively limited group of IEIs, which will be discussed below. However, a predisposition to invasive bacterial infections, including sepsis and meningitis, often in the absence of fever, can be noted in isolated congenital asplenia, complement deficiencies, antibody deficiency states, and innate immunity defects such as IRAK4 or MYD88 deficiency [3, 4].

Predominantly antibody deficiencies are the most common type of IEIs, accounting for almost half of all cases [2, 6, 28], and include, for example, X-linked agammaglobulinemia, common variable immunodeficiency, and selective IgA deficiency, which is the most common primary immunodeficiency overall [3]. In general, affected individuals are prone to recurrent pyogenic infections caused by encapsulated bacteria, and chronic enteroviral and gastrointestinal protozoal infections, especially giardiasis [2, 3, 9]. Other clinical features include autoimmune disease, such as autoimmune hemolytic anemia and idiopathic thrombocytopenic purpura, lymphoid hyperplasia or hypoplasia, and endocrine abnormalities [8]. Immunoglobulin replacement therapy (IgRT) is the cornerstone of treatment for these disorders, reducing the frequency and severity of infections and increasing life expectancy [8].

X-linked agammaglobulinemia (XLA), also known as Bruton’s agammaglobulinemia, is characterized by severe reduction of all serum immunoglobulin isotypes with markedly decreased or absent circulating B cell population, but normal numbers of pro-B cells [1, 2, 8, 9, 28]. It is caused by mutations in the Bruton’s tyrosine kinase (BTK) gene, a key regulator of early B cell maturation [9, 28].

Once the maternally derived IgG antibodies levels decrease after the first 6 to 9 months of life, patients are at risk to develop recurrent ear-nose-throat and airway infections [3, 9, 28], with only about 20% remaining asymptomatic until 3 to 5 years of age [9, 16]. Less common presentations include chronic conjunctivitis, gastrointestinal protozoal infections, CNS enteroviral infections, rheumatoid-like arthritis, and increased risk of malignancy [9, 28]. XLA patients also show increased risk of live vaccine-related poliomyelitis [16].

**Fig. 7:**
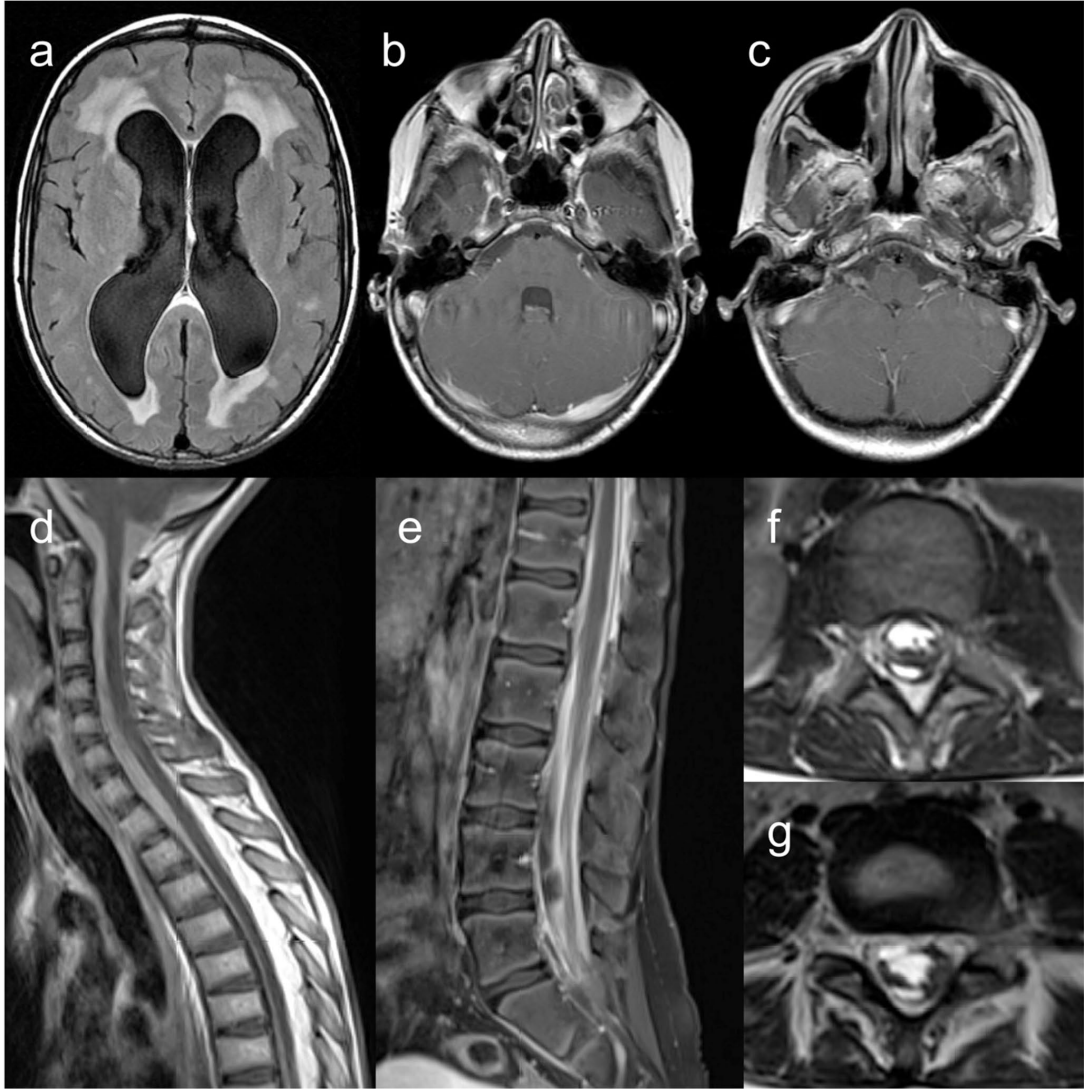
X-linked Agammaglobulinemia. A 16-year-old female patient with chronic enteroviral meningitis associated with congenital agammaglobulinemia. Brain MRI (**a** – axial FLAIR, and **b** and **c** – gadolinium-enhanced axial T1) shows chronic supratentorial hydrocephalus, which is slightly more pronounced compared to previous scans (not shown). There is concomitant transependymal edema, partially confluent with multifocal T2-hyperintense white matter lesions. Leptomeningeal enhancement is observed surrounding the brainstem, involving some cranial nerves, and extending into the internal auditory canals. Spine MRI (**d** and **e** – gadolinium-enhanced sagittal T1, and **f** and **g** – gadolinium-enhanced axial T1 at two lumbar levels) reveals features suggestive of extensive spinal pachymeningitis and arachnoiditis

CNS disease primarily results from enteroviral infections that can manifest as insidious cognitive deterioration, seizures and neurologic deficits, but also progressive myelopathy, retinopathy and sensorineural hearing loss [10, 29]. Neuroimaging shows diffuse leptomeningeal thickening and enhancement, encephalitis, and progressive cerebral atrophy in relation to chronic enteroviral infection [8, 9] (Fig. 7).

The clinical course varies from rapidly progressive, fatal encephalitis to fluctuating, chronic meningoencephalitis resulting in slow decline over as long as two decades [16]. Enterovirus infections may also disseminate into a dermatomyositis-meningoencephalitis syndrome [10, 16].

Combined immunodeficiency disorders (CIDs) are a heterogeneous group of conditions that arise from defects in various stages of T cell development or function, affecting both cellular and humoral immunity. Patients demonstrate reduced T cell function, which is absent in cases of severe combined immunodeficiency (SCID). The number of circulating B cells is typically normal, though impaired or absent B cell and antibody function often coexists. CIDs have different clinical phenotypes and may present with associated or syndromic features that serve as diagnostic clues [1, 4, 8].

Immune dysfunction has been reported in a few genetic disorders related to vitamin B12 and folate metabolism, such as transcobalamin deficiency and methylenetetrahydrofolate dehydrogenase, cyclohydrolase and formyltetrahydrofolate synthetase 1 (MTHFD1) deficiency [30]. In contrast to adenosine deaminase (ADA) deficiency, in which defective purine synthesis results in SCID, MTHFD1 deficiency highlights the importance of de novo thymidylate biosynthesis for lymphocyte proliferation and immune function [31]. Unlike these inborn errors of metabolism, acquired vitamin B12 deficiency generally results in only mild immune dysfunction and is often manageable with supplementation.

Wiskott-Aldrich syndrome (WAS) is an X-linked recessive combined immunodeficiency with congenital thrombocytopenia caused by defects in WAS protein, a regulator of actin filament reorganization [1, 8, 9, 20]. It is marked by a progressive decrease in T cells numbers and abnormal lymphocyte responses to anti-CD3 [1, 32]. Patients exhibit normal B cell numbers but have low serum IgM levels, often elevated serum IgA and IgE levels, and impaired antibody responses to polysaccharide antigens, predisposing to infections caused by common polysaccharide-encapsulated pathogens, but also by opportunistic microorganisms, including cytomegalovirus, *Pneumocystis*, and *Aspergillus* [1, 9, 32].

The wide clinical spectrum of WAS ranges from isolated, mild thrombocytopenia with small platelets to life-threatening hemorrhage in about 30% of patients, eczematous dermatitis, combined immunodeficiency with severe recurrent sinopulmonary infections, autoimmune disease (autoimmune hemolytic anemia, IgA nephropathy, skin vasculitis, and chronic arthritis) and neoplasms, most commonly lymphoproliferative disorders and myelodysplasia [1, 8, 9, 28, 32, 33]. Some unique features are necrotizing vasculitis and aneurysmal arterial dilatation [28, 33]. Massive bleeding, infection, and lymphoreticular malignancies are the major causes of mortality in these patients [9].

**Fig. 8:**
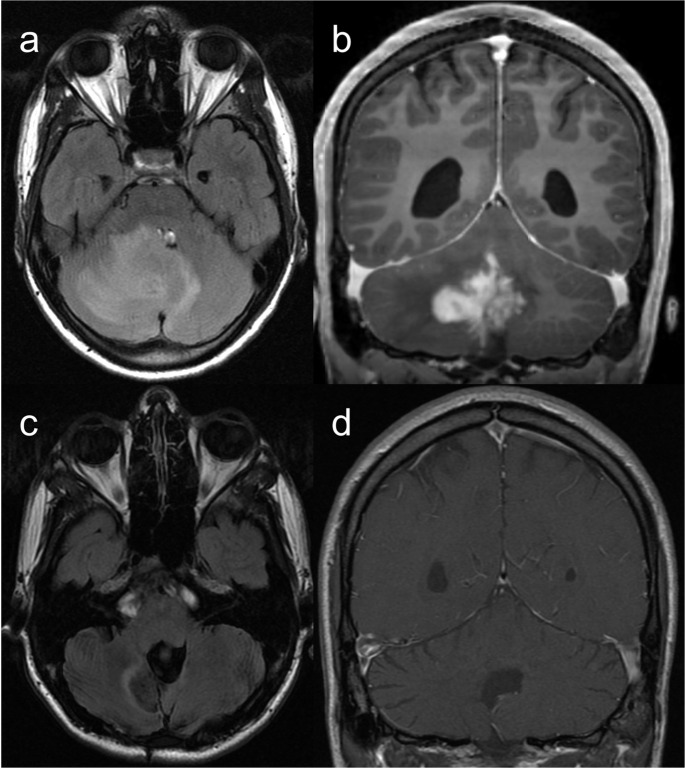
Wiskott-Aldrich Syndrome. A 20-year-old male patient with mild thrombocytopenia characterized by small platelets and autoimmune thyroiditis, presenting with a progressive and severe occipital headache. Initial brain MRI (**a** – axial FLAIR, and** b** – gadolinium-enhanced coronal T1) demonstrates an infiltrative enhancing cerebellar lesion, with no signs of CSF dissemination. The differential diagnosis includes EBV-associated lymphoproliferative disorder or lymphoma, considering the patient’s underlying condition, or another high-grade tumor of the CNS. CSF analysis was negative for neoplastic cells, and histopathology revealed a non-neoplastic, non-specific inflammatory lesion, characterized by a population of small T lymphocytes, which excluded malignancy (negative for herpes virus, cytomegalovirus, SV40, and EBV). Follow-up brain MRI 9 months post-surgery (**c** – axial FLAIR, and **d** – gadolinium-enhanced coronal T1) shows post-surgical changes in the posterior fossa, with complete resection of the mass and no signs of recurrence

Reported neuroimaging findings in WAS are largely limited to case reports of extranodal non-Hodgkin lymphoma and intracranial hemorrhage [33] (Fig. 8). Diffuse cerebral microhemorrhages have also been documented, with thrombocytopenia and autoimmune small vessel vasculitis being proposed as potential pathogenic mechanisms [33].

Congenital defects of phagocyte number or function predispose individuals to severe, recurrent pyogenic bacterial and fungal infections, as well as infections caused by intracellular microorganisms such as mycobacteria and *Nocardia* [2, 3, 9]. Increased susceptibility to viral and protozoal infections or an increased risk of malignancy are not associated features of this group of diseases [9].

Chronic granulomatous disease (CGD), one of the most common phagocyte disorders, results from defective respiratory burst due to abnormalities in nicotinamide adenine dinucleotide phosphate (NADPH) oxidase, which is responsible for superoxide generation in phagocytes [1, 8–10, 28]. As a result, the clinical presentation is characterized by a marked susceptibility to infections caused by catalase-positive bacteria and fungi [2, 3, 8, 9]. Symptoms usually appear within the first year of life [9] and include recurrent, life-threatening infections of the lungs, skin, lymph nodes, and other internal organs [9], along with granuloma formation [8, 16]. The leading cause of death in these patients is invasive aspergillosis [28].

**Fig. 9:**
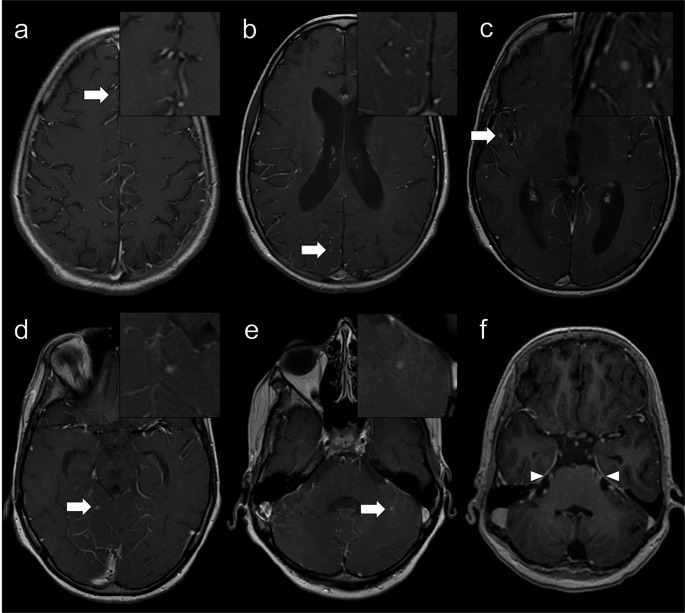
Chronic granulomatous disease. A 16-year-old male patient with meningitis caused by *Granulibacter bethesdensis* and *Rhizobium radiobacter*. The patient has a history of lymphadenitis, pneumonia, and sepsis without an identified causative agent. Gadolinium-enhanced axial T1-weighted images reveal multiple nodular enhancing lesions in the cerebral and cerebellar subcortical regions (arrows), without low signal on ADC maps (not shown), likely representing microabscesses. There is also leptomeningeal enhancement, most pronounced in the posterior fossa involving the medulla and trigeminal nerves (arrowheads), and mild supratentorial hydrocephalus

Brain abscesses have been described in association with various pathogens [10, 16, 28, 34] including *Granulibacter bethesdensis*, an emerging microorganism that causes infection exclusively in CGD patients [35] (Fig. 9). Other reported neurologic complications in CGD include CNS granulomatous disease, white matter disease, and leptomeningeal and brain infiltration by pigmented lipid-laden histiocytes [10, 34].

Additionally, CNS infectious disease is the hallmark of some defects in intrinsic and innate immunity.

Herpes simplex encephalitis (HSE) is the dominant clinical phenotype during primary infection with *Herpes simplex virus* (HSV) type 1, typically between 3 months and 6 years of age, in patients with mutations in genes encoding Toll-like receptor 3 (TLR3) or other TLR pathway members [1, 3, 4, 15].

**Fig. 10:**
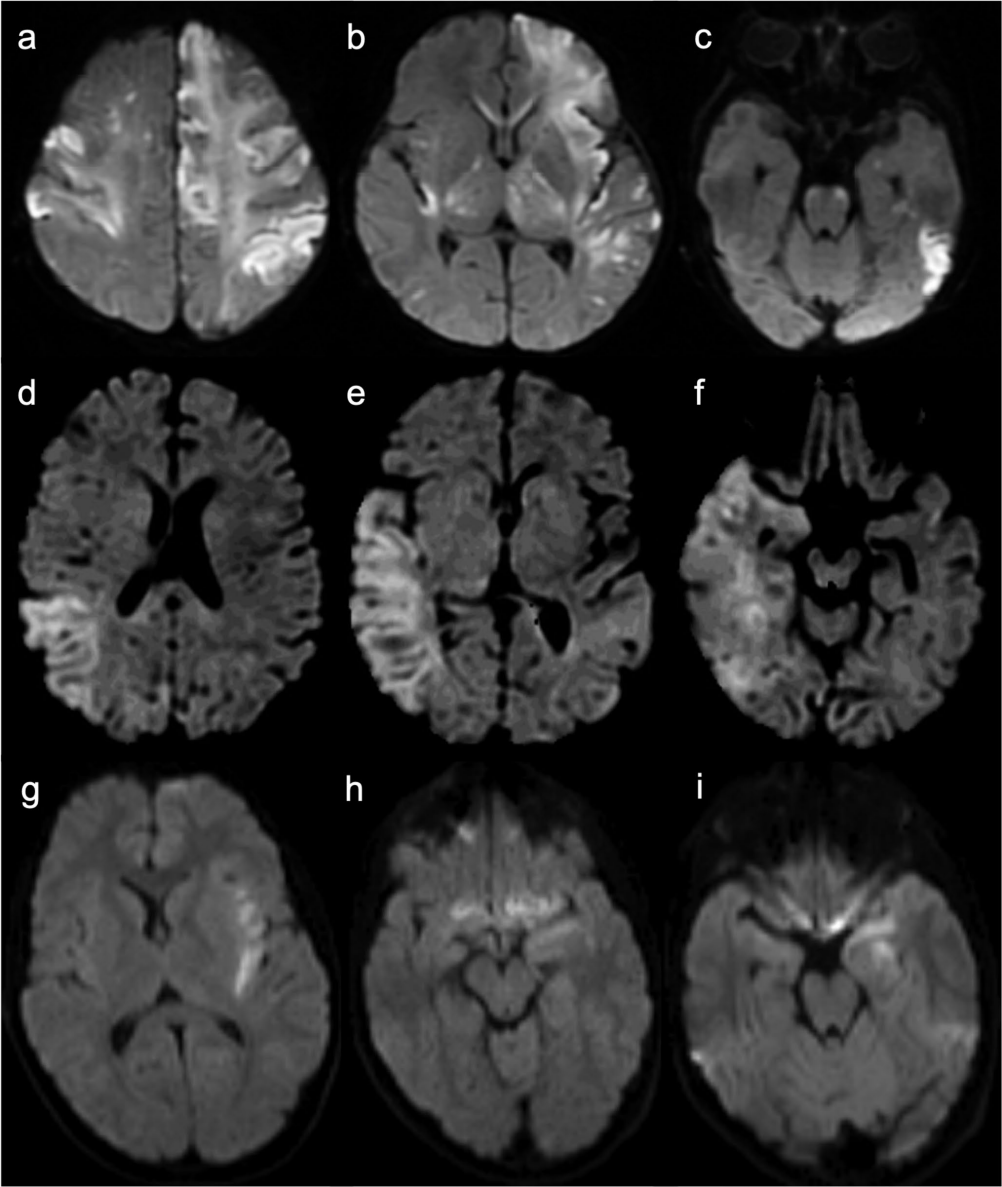
Diffusion MR imaging patterns in Herpes simplex encephalitis. (**a** to **c**) Neonatal HSV type 2 encephalitis in a 20-day-old male patient. Multifocal, asymmetric areas of DWI hyperintensity (corresponding low signal on ADC maps is not shown) involving cortical and subcortical regions, including the corpus callosum, thalami, posterior limbs of the internal capsules, and anterior pons. (**d** to **f**) HSV type 1 encephalitis in a 12-month-old female patient. Bilateral asymmetric cortico-subcortical lesions with DWI hyperintensity, predominantly in the right temporoparietal region, mimicking a right middle cerebral artery infarction. (**g** to **i**) HSV type 1 encephalitis in an 11-year-old male patient. Predominantly left-sided cortical lesions with DWI hyperintensity involving the medial temporal lobe, insula, and frontal cortex; subtle frontobasal involvement on the right. Cortical hemorrhagic foci also present (not shown)

Neonatal HSE is most often caused by HSV type 2 infection acquired during or shortly after birth, typically presenting between 2 and 4 weeks of age. It is highly lethal and exhibits an imaging pattern distinct from its more common non-neonatal counterpart. Unlike the predominant involvement of the medial temporal and inferior frontal lobes, insula, and cingulate gyrus seen in HSV type 1 encephalitis in adults and older children, neonatal HSE diffusely affects the periventricular white matter, cerebral cortex, and thalami, and petechial hemorrhage is rare [36, 37]. In contrast, MR imaging in HSV type 1 encephalitis in infants and young children under two years of age usually shows cortical and adjacent white matter abnormalities with a vascular distribution in the parietal, occipital, and temporal lobes, which suggests hematogenous spread of the virus in this age group, rather than along meningeal branches of the trigeminal ganglion (Fig. 10) [37–39].

HSE and other viral infections of the brainstem are characteristic of autosomal recessive DBR1 deficiency [1, 3]. HSE may also occur in other IEIs such as NEMO deficiency, STAT1 deficiency, CVID, DOCK8 or GATA2 deficiency [3]. Mollaret’s meningitis, or recurrent lymphocytic meningitis due to HSV type 2, has been reported in patients with SNORA31, ATG4A, or MAP1LC3B2 deficiency [1].

**Fig. 11:**
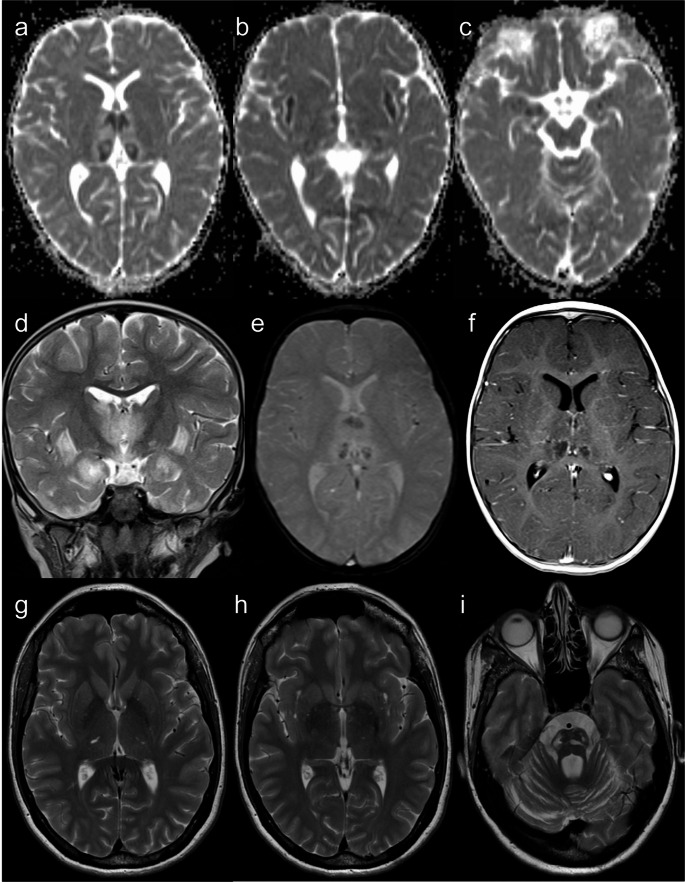
Familial acute necrotizing encephalopathy. A 13-month-old male patient with status epilepticus, preceded by fever and lethargy. His brain MRI (**a** to **c** – axial ADC maps, **d** – coronal T2, **e** – axial SWAN, and **f** – gadolinium-enhanced axial T1) demonstrates symmetrical T2-hyperintense lesions in the thalami, external capsules, and hippocampi. These lesions exhibit low signal on ADC maps, hemorrhagic foci, and thin peripheral enhancement. Mass effect is noted, leading to compression of the third ventricle and the temporal poles of the lateral ventricles. Overall, these findings are consistent with ANE. The patient’s mother had an episode described as corticosteroid-responsive encephalomyelitis with elevated anti-Influenza B antibody titers at 12 years of age. Her brain MRI at 40 years of age (**g** and **i** – axial T2) shows cavitated lesions in the thalami, sublenticular regions, external capsules, and ventral pons bilaterally, accompanied by secondary diffuse cerebellar atrophy. These findings are compatible with sequelae of ANE. Genetic testing confirmed the presence of RANBP2 gene mutations in both the patient and his mother

Recurrent and/or familial episodes of acute necrotizing encephalopathy (ANE) secondary to viral infections, most commonly influenza, are linked to autosomal dominant mutations in the RANBP2 gene in both pediatric and adult populations [1, 3, 4] (Fig. 11).

CARD9 deficiency affects mononuclear phagocytes and predisposes to invasive fungal disease that may affect the CNS [1, 3].

#### Inflammatory

Hemophagocytic lymphohistiocytosis (HLH) is a severe multisystem disorder of immune dysregulation and hyperinflammation, often triggered by herpesvirus infections [1, 16, 40, 41]. Clinical and laboratory diagnostic criteria include prolonged high fever, splenomegaly, cytopenias, low or absent NK cell activity, hypertriglyceridemia and/or hypofibrinogenemia, and hyperferritinemia. These features result from the diffuse infiltration of lymphoid tissue and other organs by polyclonal activated T cells and macrophages, which secrete large amounts of inflammatory cytokines, in both inherited and acquired forms of HLH. The latter is secondary to severe infections in immunocompromised patients or occurs as a complication of malignancies or rheumatic diseases [16, 40, 41].

Different IEIs can cause HLH, namely X-linked lymphoproliferative disease types 1 and 2, familial hemophagocytic lymphohistiocytosis (FHLH) syndromes, and FHLH syndromes with oculocutaneous hypopigmentation: Chédiak-Higashi syndrome (CHS), Griscelli syndrome type 2, HPS types 2 and 10, and CEBPE neofunction [1, 16]. HLH is associated with other inherited diseases of immune dysregulation such as CD27 deficiency, and autoinflammatory disorders, in particular CDC42 deficiency and T cell lymphoma subcutaneous panniculitis-like/TIM3 deficiency [1].

CNS involvement occurs in up to 70% of patients, and it is one of the main causes of death in HLH, in addition to hemorrhage and sepsis. However, if detected early, it may be treatable [16].

Polyclonal activated T cells and macrophages infiltration begin in the meninges and progresses through perivascular spaces with subsequent diffuse tissue involvement, mainly affecting the white matter. Multifocal demyelination and necrosis may follow [16, 40]. Neuroimaging plays crucial role in early detection and accurate evaluation of the severity of CNS involvement in HLH, showing a good correlation with clinical findings. Additionally, elevated levels of neopterin in the CSF and soluble interleukin-2 receptor alpha-chain (sCD25) in the serum are considered good markers of CNS disease [41].

Initial brain MRI abnormalities include multiple T2 hyperintense cerebral or cerebellar lesions with nodular or ring enhancement, with a predominant distribution at the grey-white matter junction, and less frequently involving the brainstem. These lesions can become confluent, sometimes with hemorrhagic transformation. Low signal on ADC maps corresponding to the enhancing lesions has been reported during the active phase [16, 41].

**Fig. 12:**
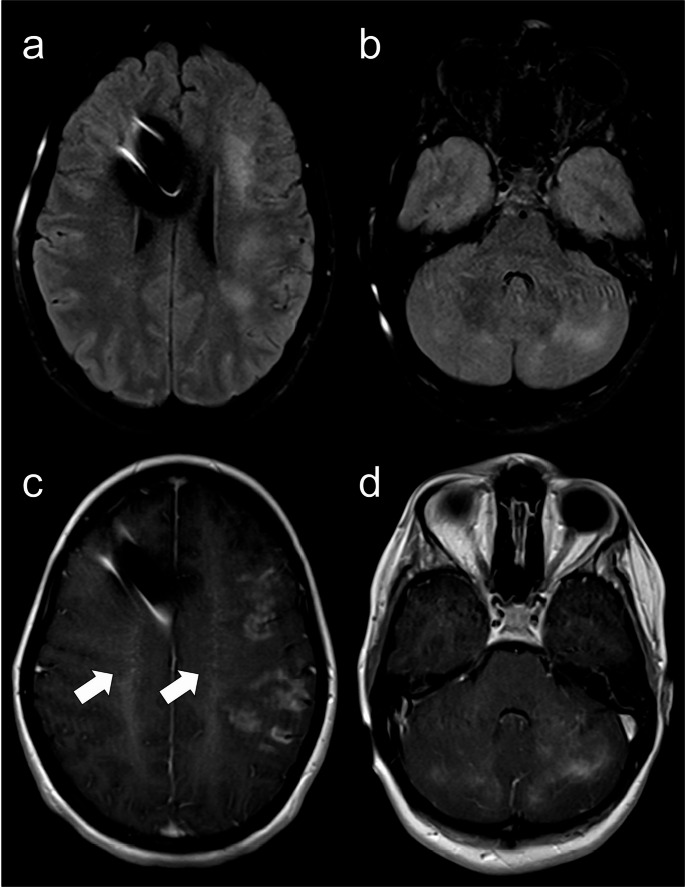
Hemophagocytic lymphohistiocytosis. A 23-year-old female patient with refractory septic shock in the context of hemophagocytic lymphohistiocytosis with predominant CNS manifestations. Genetic testing identified a homozygous variant with uncertain clinical significance in the UNC13D gene, associated with familial HLH. Brain MRI (**a** and **b** – axial FLAIR, and **c** and **d** – gadolinium-enhanced axial T1) demonstrates bilateral tumefactive, partly confluent, T2-hyperintense cortico-subcortical lesions with heterogeneous patchy enhancement, involving both supra and infratentorial regions. Prominent perivascular enhancement is noted on the centra semiovalia (arrows on c). A frontal ventriculoperitoneal shunt catheter is present

Other neuroimaging findings include leptomeningeal and perivascular enhancement (Fig. 12), mild ventriculomegaly due to communicating hydrocephalus, subdural collections, cortical laminar necrosis, and diffuse brain edema. Over time, parenchymal atrophy ensues [41]. Although non-specific, MR spectroscopy can be useful in assessing inflammatory activity. It may reveal a lactate peak and elevated glutamine/glutamate levels during the acute phase, and show an elevated choline peak with a reduced N-acetylaspartate peak reflecting ongoing tissue destruction [41].

While neurologic manifestations associated with HLH typically arise in the first years of life, patients with CHS who survive into adulthood develop a chronic neurodegenerative disease with progressive cerebral and cerebellar atrophy, dementia, ataxia, autonomic dysfunction, and parkinsonism (7,16).

X-linked lymphoproliferative disease type 1 (XLP1) is an X-linked recessive disorder of immune dysregulation with susceptibility to Epstein-Barr virus (EBV) infection and lymphoproliferative conditions caused by pathogenic variants in the SH2D1A gene, which encodes the signaling lymphocyte activation molecule associated protein (SAP) expressed in T cells, NK cells, and invariant NK cells [1, 16, 42].

Affected males usually present with clinical and immunologic features triggered by EBV infection including fulminant mononucleosis, HLH, hypogammaglobulinemia, and lymphoma. Rarer manifestations include bone marrow failure with aplastic anemia, and CNS vasculitis [1, 16, 42]. Median age at presentation is 3–4 years, and only about half of the patients survive into adulthood [42].

**Fig. 13:**
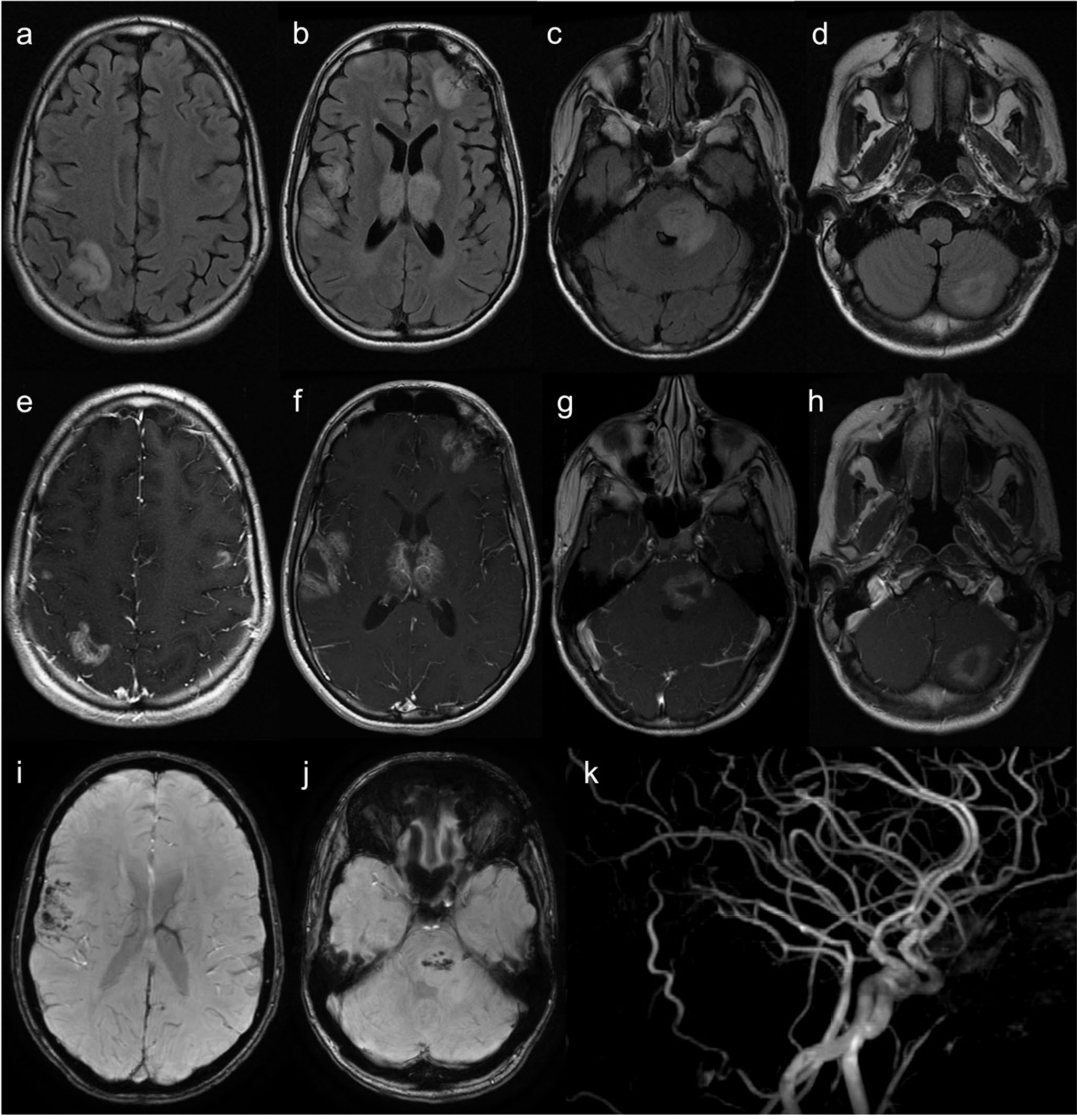
X-linked lymphoproliferative disease type 1. A 16-year-old male with behavioral changes and a history of intestinal Burkitt lymphoma resected two years earlier. Brain MRI (**a** to **d** – axial FLAIR, **e** to **h** – gadolinium-enhanced axial T1, and **i** and **j** – axial SWAN) shows multifocal edematous supra and infratentorial lesions, predominantly affecting the cortico-subcortical regions, with heterogeneous enhancement and some hemorrhagic foci. (**k**) No abnormalities were detected in the 3D reconstruction of the TOF MR angiography. Despite the bithalamic involvement with hemorrhage, also seen in parainfectious ANE (Fig. 11), the progressive clinical course (not depicted), presence of multiple asymmetric cortico-subcortical lesions, intense enhancement, and sparing of the internal and external capsules, and medial temporal regions are, in this case, more consistent with CNS vasculitis. This diagnosis was confirmed by brain biopsy, which excluded lymphoma

CNS vasculitis is mostly found in association with EBV infection, but it can also occur in its absence [16, 42, 43] (Fig. 13). Most cases of vasculitis occur in late childhood and adolescence, with a median age of 16 years [42]. Despite immunosuppression, the prognosis of XLP1-related CNS vasculitis is usually poor [42]. Yet, hematopoietic stem cell transplant (HSCT) can be curative, even in patients with CNS involvement [16].

While CNS vasculitis is not uncommon in XLP1, it is rarely found in patients with X-linked lymphoproliferative disease type 2 (XLP2), which results from mutations in the XIAP gene [7].

Vasculitis of the CNS has also been reported in other IEIs including AR-HIES due to DOCK8 deficiency [7, 25], Blau syndrome, chronic mucocutaneous candidiasis and immunodeficiency [7], purine nucleoside phosphorylase (PNP) deficiency [11], and common variable immunodeficiency [10].

Common variable immunodeficiency disorders (CVID) comprise several clinical and laboratory phenotypes that may be caused by various genetic defects and/or environmental factors. As a group, primary CVID represents one of the most common symptomatic forms of IEIs, usually in the second or third decades of life [1, 3, 15, 44]. Due to abnormal B cell differentiation or defective T/B cell interactions, hypogammaglobulinemia and abnormal antibody formation in response to vaccines or other antigens are characteristic. Consequently, the main clinical features include recurrent bacterial respiratory and gastrointestinal infections, impaired vaccination response, and an increased incidence of autoimmune manifestations and malignancies [15, 44].

Although CNS involvement in CVID and CVID-like patients is typically rare, it has been documented as a symptom of severe systemic immune dysregulation, presenting as acute disseminated encephalomyelitis, autoimmune limbic encephalitis, or granulomatous disease. The neuroimaging spectrum of inflammatory lesions in CVID includes encephalitis, vasculitis, granulomatous meningitis, myelitis, and radiculitis [15]. Peripheral nervous system involvement has also been reported with cases of chronic inflammatory demyelinating polyneuropathy [12].

Infectious CNS disease is rare in these patients and is mostly limited to rare viral infections post-IgRT, including progressive multifocal leukoencephalopathy caused by John Cunningham virus (JCV) [12, 15].

Neonatal onset multisystem inflammatory disorder (NOMID), also known as chronic infantile neurological cutaneous and articular (CINCA) syndrome, is an autosomal dominant autoinflammatory disease that results from gain-of-function mutations in the NLRP3 gene [1, 7]. Patients typically exhibit a neonatal onset nonpruritic rash, continuous fever, secondary amyloidosis, deforming arthropathy, and prominent CNS involvement [1, 7, 44]. Chronic aseptic meningitis, present in almost all cases, is the major cause of morbidity and mortality [7]. Optic disc pathology is frequently observed, ranging from papilledema and papillitis to optic nerve atrophy with visual loss. The proposed underlying mechanisms include primary optic neuritis, infiltrative optic neuritis due to chronic aseptic meningitis, and increased intracranial pressure [44]. Other common neurologic features are developmental delay, progressive sensorineural hearing loss, and seizures [4, 7].

#### Neurodegenerative

Ataxia-telangiectasia (AT) is an autosomal recessive disorder caused by mutations in the ATM gene, which encodes a DNA-dependent kinase involved in DNA repair mechanisms [8]. It is primarily characterized by progressive cerebellar ataxia, oculocutaneous telangiectasias, combined immunodeficiency with recurrent ear-nose-throat and bronchopulmonary infections, and increased risk of lymphoreticular and other neoplasms. Associated features include increased serum alpha-fetoprotein levels, and radiosensitivity, chromosomal instability, and chromosomal translocations due to impaired DNA repair [1, 7, 11].

The early clinical picture is dominated by cerebellar dysfunction due to the widespread neurodegeneration of Purkinje cells [7, 10]. Head and truncal ataxia typically develop by 3 years of age, progressively worsening with gait abnormalities [7, 11]. Basal ganglia dysfunction emerges in the later stages of the disease, manifesting as hypotonia, tremor, and choreoathetosis [10]. Abnormal eye movements are also characteristic, and peripheral neuropathy with axonal degeneration may occur by the third decade of life [14, 16].

Neuroimaging studies reveal progressive cerebellar atrophy, first involving the superior vermis and lateral aspects of the cerebellar hemispheres [7–9, 11, 12]. Other findings include lymphoid and thymic hypoplasia, and recurrent middle ear and sinonasal infections [9].

Ataxia-telangiectasia-like disorder (ATLD) is a genomic instability syndrome that shares phenotypic features with AT but is caused by homozygous or compound heterozygous MRE11A gene mutations [45]. Affected patients do not show telangiectasias, immunodeficiency is mild or absent, and the neurologic symptoms have a later onset and slower progression than in AT [7].

Aicardi-Goutières syndrome (AGS) is a progressive inflammatory encephalopathy caused by mutations in seven genes encoding intracellular molecules involved in nucleic acid metabolism or signaling, which ultimately leads to chronic activation of type I interferon [1, 14, 46]. Early-onset neurodegeneration and defective myelination are responsible for the neurologic symptoms [7, 46]. The neonatal form mimics congenital viral infections (pseudo-TORCH) with spasticity, dystonia, seizures, and neurodevelopmental delay. In the less common late-onset form, initial normal psychomotor development is followed by subacute regression, extreme irritability, periodic fever, and progressive microcephaly [10, 14, 46]. Death often occurs in early childhood [13, 46].

**Fig. 14:**
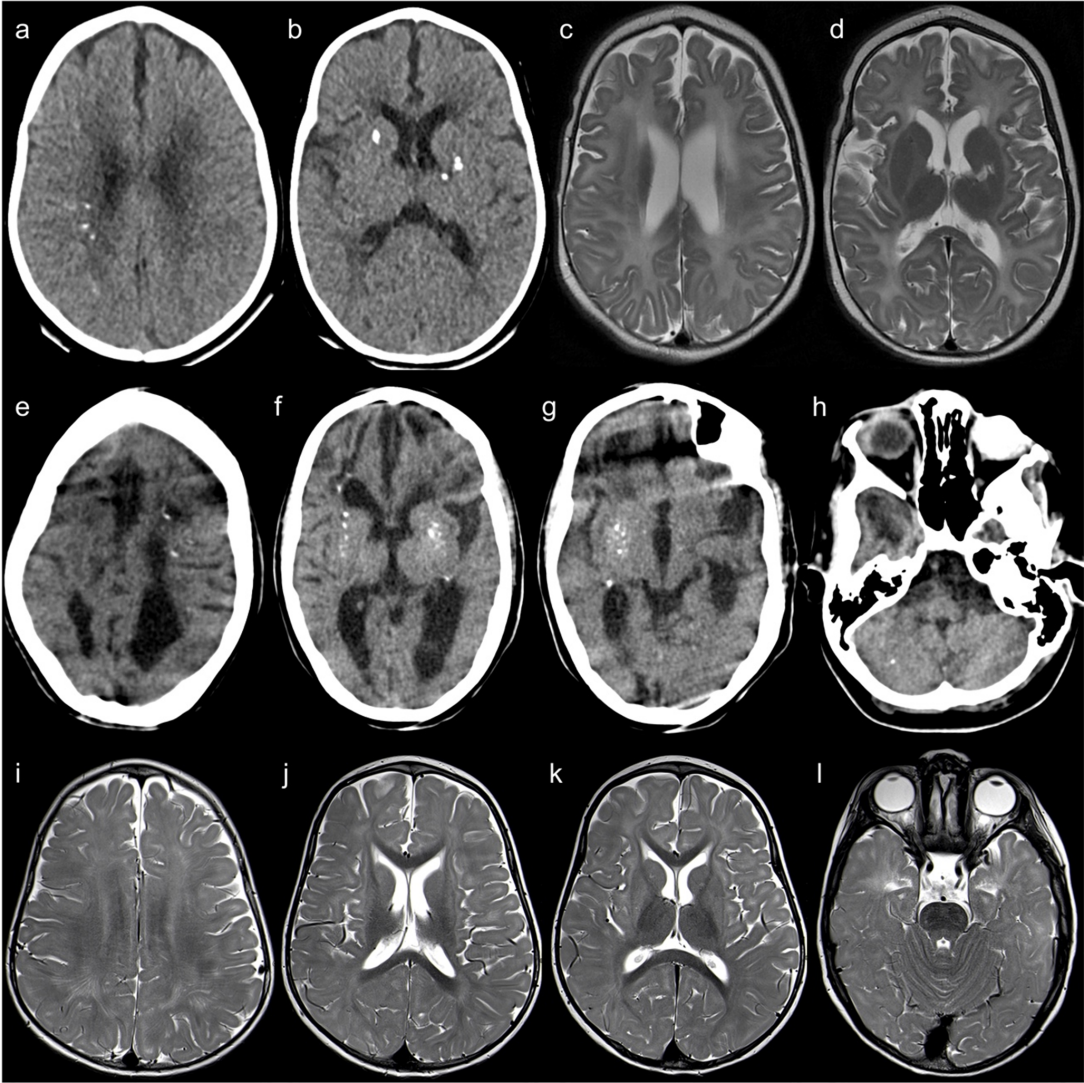
Different neuroradiologic patterns in Aicardi-Goutières syndrome. AGS type 5 (SAMHD1 mutations). An 8-month-old male patient with low birth weight, developmental delay, lower limb hypertonia, and axial hypotonia. Head CT scan (**a** and **b**) shows multiple spot-like calcifications in the basal ganglia, internal capsules, coronae radiatae, and right frontoparietal deep white matter. Brain MRI (**c** and **d** – axial T2) reveals predominantly periventricular leukoencephalopathy and a left basal ganglia sequelae, probably of vascular etiology. AGS type 5 (SAMHD1 mutations). A 9-year-old female patient with severe prenatal encephalopathy, epilepsy treated with vigabatrin, and moderate cognitive deficit. Head CT scan (**e** to **h**) shows millimetric spot-like calcifications in the basal ganglia, cerebral white matter, and right cerebellar hemisphere. There is also diffuse brain atrophy and leukoencephalopathy with multifocal hypodensities of the residual white matter and apparent frontal subcortical cystic areas. AGS type 6 (ADAR1 mutations). A 3-year-old patient with developmental regression, hypotonia, and macrocephaly. (**i **to** l**) Axial T2 images demonstrate bilateral striatal T2-hyperintense lesions and diffuse leukoencephalopathy

The classic features of AGS - brain calcifications, leukoencephalopathy, and cerebral atrophy - are present in almost all cases [46] and are considered virtually pathognomonic [26]. Nonetheless, the neuroimaging patterns are heterogeneous, reflecting the complex pathogenesis and timing of disease onset [46] (Fig. 14).

Calcifications are usually symmetrical, spot-like, and located in the basal ganglia, particularly the lentiform nuclei, and the frontoparietal deep white matter. Other areas of involvement include the dentate nuclei, brainstem, and both the cerebellar and cerebral cortices [26, 46]. True periventricular calcifications are rare [26]. If the clinical presentation is typical, a single calcification may be enough to suggest the diagnosis [46]. Severe calcification is associated with mutations in the TREX1 gene (AGS type 1) and an age of onset of less than 3 months; however, it is inversely related to AGS type 2 caused by mutations in the RNASEH2B gene [46].

There are three main patterns of leukoencephalopathy in AGS: frontotemporal predominance, periventricular predominance, and diffuse involvement. A strong association has been demonstrated between frontotemporal white matter rarefaction and both AGS type 1 and an age at onset of less than 3 months [46].

Delayed myelination is seen in almost one-third of the patients. Cerebral atrophy is attributed to white matter loss, with relative sparing of the cortex, but improvement can be seen in some patients. Basal ganglia atrophy is present in cases of bilateral striatal necrosis, especially in ADAR1 deficiency (AGS type 6) [1, 26, 46].

Hoyeraal-Hreidarsson syndrome (HHS) is a multisystem genetic disorder associated with aberrant telomere biology [10, 16, 47]. To date, causative autosomal dominant mutations in TINF2 gene, autosomal recessive mutations in TERT, ACD and RTEL1 genes, and X-linked recessive mutations in DKC1 gene have been identified [1, 47]. HHS is a severe form of DKC, but the mucocutaneous triad of nail dysplasia, reticular skin pigmentation and oral leukoplakia is not always present at diagnosis [16, 47]. Patients typically present with intrauterine growth retardation, microcephaly, cerebellar hypoplasia, progressive bone marrow failure and severe combined immunodeficiency [10, 14, 47–49]. Other clinical manifestations include pulmonary and liver fibrosis, esophageal dysfunction, stenosis of the lacrimal ducts and urethra, avascular joint necrosis, and premature greying of the hair [47]. Associated CNS symptoms also include spastic paresis, peripheral demyelinating neuropathy, and seizures [16, 47].

**Fig. 15:**
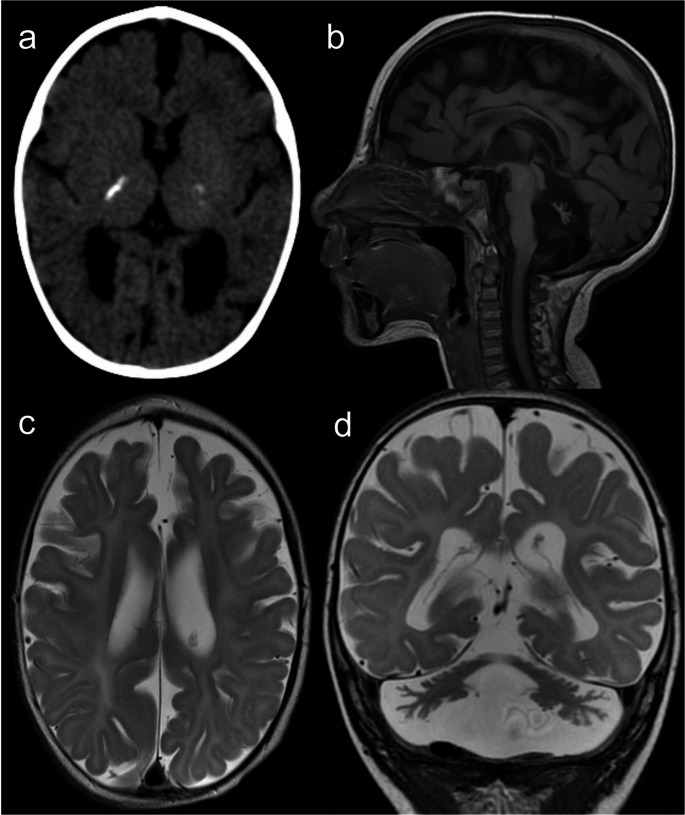
Hoyeraal-Hreidarsson syndrome. An 8-month-old male patient with microcephaly, developmental delay, and oral and skin lesions. Head CT (**a**) and brain MRI (**b** – sagittal T1, **c** – axial T2, and **d** – coronal T2) scans depict prominent pontocerebellar hypoplasia and coarse calcifications in the internal capsules. Other abnormalities include discrete T2 hyperintensity of the cerebral white matter, hypoplastic corpus callosum, and microcephaly with a simplified gyral pattern. These imaging findings are suggestive of Hoyeraal-Hreidarsson syndrome, confirmed by targeted genetic testing (RTEL1 mutations)

Neuroimaging findings comprise (ponto)cerebellar hypoplasia, now considered a diagnostic requisite [47], delayed myelination, hypoplastic corpus callosum, small pituitary gland, scattered subcortical calcifications, and non-specific focal T2 hyperintensities in the brainstem and thalamus [16, 47–49]. In contrast to certain pontocerebellar hypoplasias (particularly type 2, and types 1 and 9 in some cases), cerebellar disruption due to extreme prematurity, or some neurometabolic disorders such as congenital disorder of glycosylation type 1a, which exhibit a characteristic “dragonfly” appearance of the cerebellum (i.e., marked atrophy of the hemispheres with relative sparing of the vermis), cerebellar atrophy in HHS is diffuse [49–51], as demonstrated in Fig. 15.

In the appropriate clinical context, HHS must be considered in the differential diagnosis of pontocerebellar hypoplasias. The presence of concurrent parenchymal calcifications is helpful in a neuroimaging pattern recognition-based approach and should guide targeted genetic investigations [49].

**Fig. 16:**
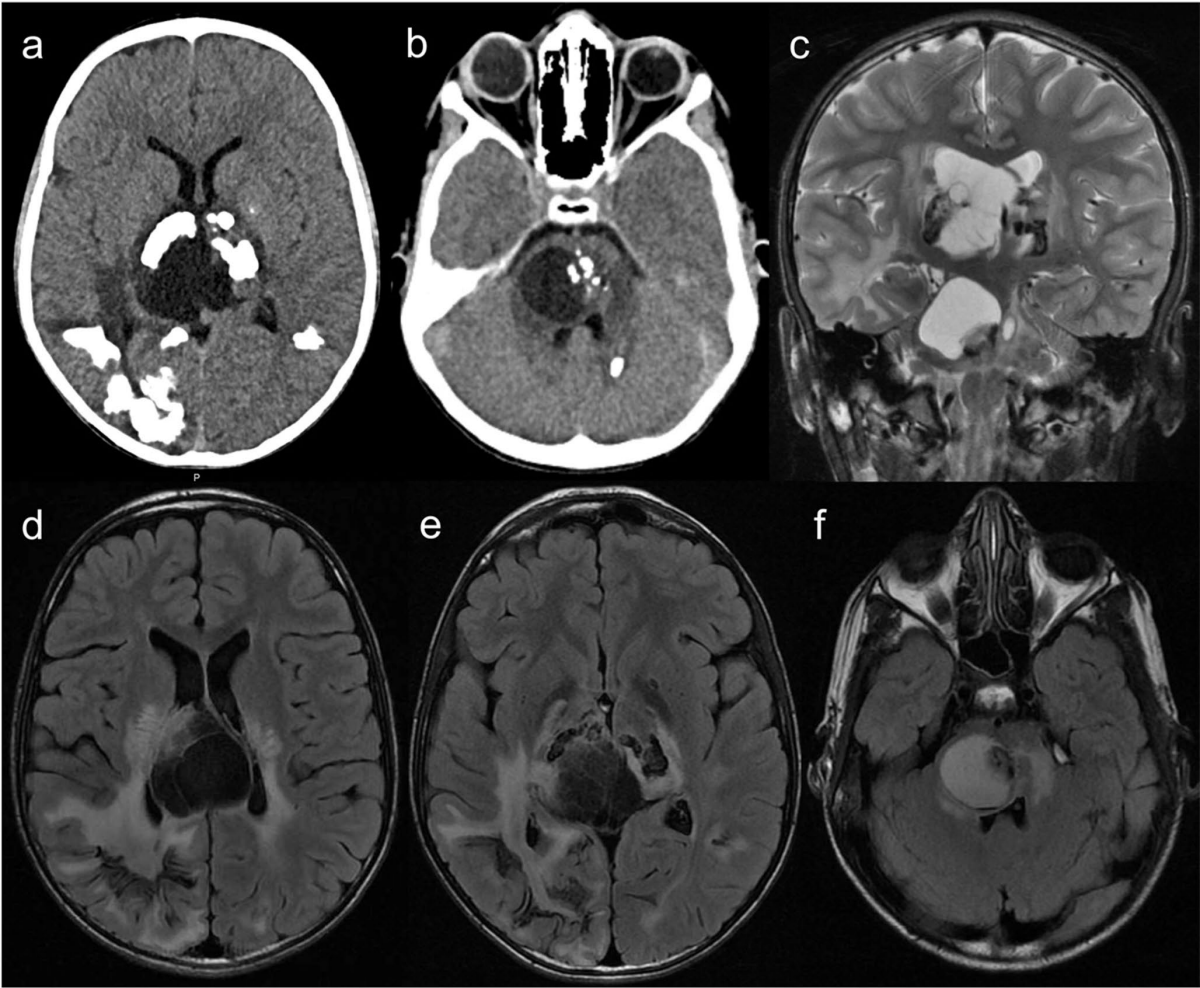
Cerebroretinal microangiopathy with calcifications and cysts. A 13-year-old male patient with asymmetric spastic tetraparesis, bilateral dysmetria, and right-sided amaurosis. Head CT scan (**a** and **b**) and brain MRI (**c** – coronal T2, and **d** to **f** – axial FLAIR) reveal coarse calcifications in the subcortical white matter, basal nuclei, and cerebellum. Intra-axial cysts are observed in the pons and right thalamus, with progressive growth (previous scans not shown), compressing the adjacent ventricular system but without hydrocephalus; the pontine cyst located exhibits higher signal than CSF on FLAIR, reflecting high protein content. There is also extensive T2 hyperintensity in the white matter, particularly around the cysts

However, one must remember that multiple intracranial calcifications can expand the differential diagnosis to include congenital TORCH infections, AGS, and cerebroretinal microangiopathy with calcifications and cysts (CRMCC) or Coats-plus syndrome, another telomere-related IEI caused by autosomal recessive inherited variants in the STN1 or CTC1 genes [1, 26, 47] (Fig. 16).

Other IEIs that may show intracranial calcifications include SPENCDI, previously discussed, and chronic atypical neutrophilic dermatosis with lipodystrophy and elevated temperature (CANDLE), caused by PSMB8 gene mutations and characterized by intracranial calcifications, contractures, panniculitis, and fever. A similar CANDLE phenotype is seen with compound heterozygous variants in the PSMB4, PSMB9, PSMA3, and POMP genes. Moreover, ISG15 deficiency is associated with susceptibility to mycobacterial infections and brain calcifications [1, 4].

#### Malignant

**Fig. 17:**
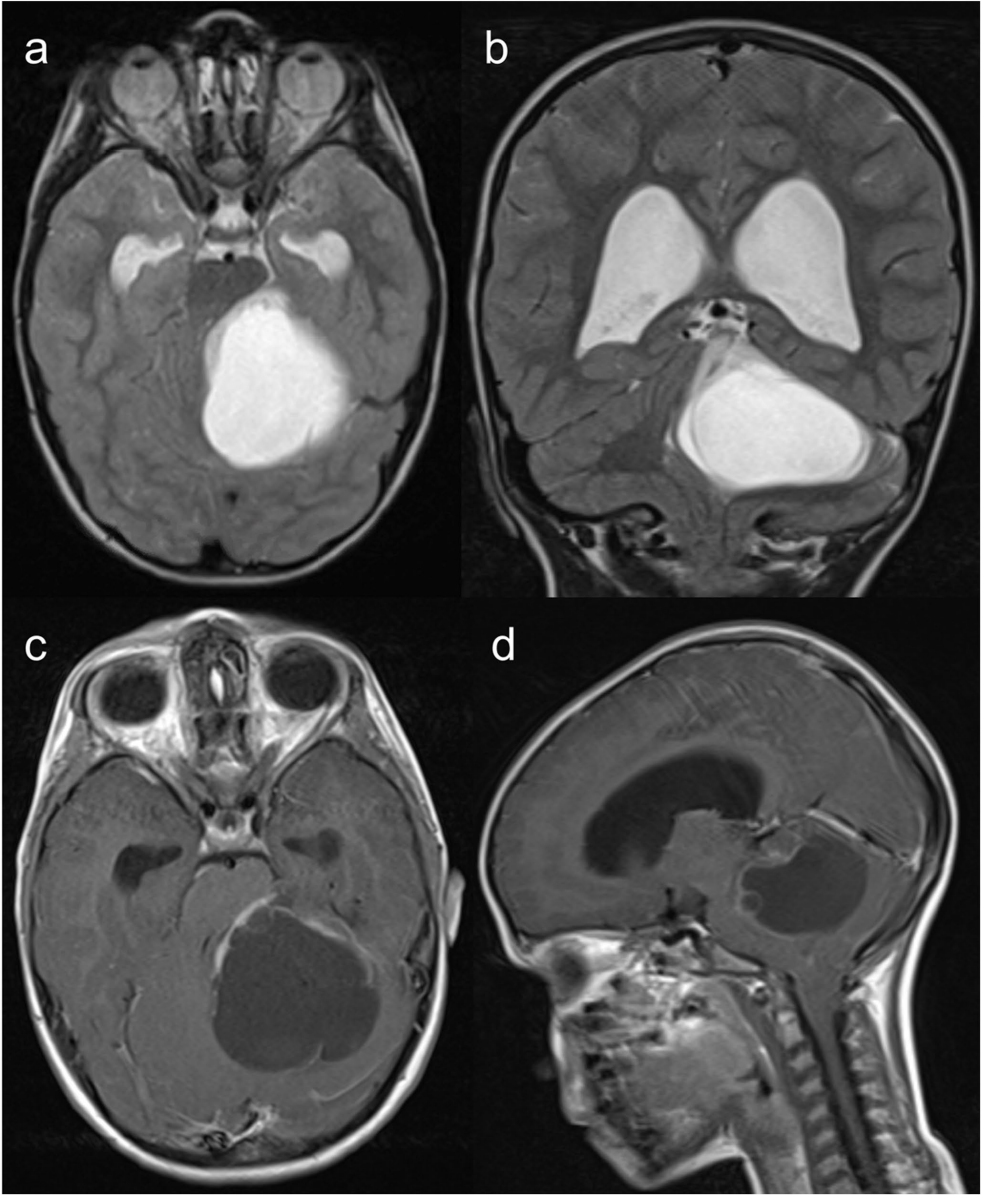
Cerebellar pilocytic astrocytoma. A 5-year-old male patient with a suspected DNA repair defect and recurrent respiratory and middle ear infections. His family history is significant for a younger brother who died at the age of 18 months from a respiratory infection, presumably related to an undetermined IEI. Brain MRI (**a **and **b** – axial and coronal T2, and **c** and **d** – gadolinium-enhanced axial and sagittal T1) shows a predominantly cystic lesion in the left cerebellum with small enhancing mural components. This lesion compresses the fourth ventricle, leading to obstructive hydrocephalus with mild transependymal edema and subtle tonsillar herniation. These findings are consistent with a pilocytic astrocytoma, which was confirmed histologically after surgical resection. Although several IEIs are associated with an increased risk of malignancy, this tumor is the second most common pediatric posterior fossa tumor and typically occurs sporadically, suggesting it may be unrelated to the suspected primary immunodeficiency in this case

Increased risk of malignancy has been described in several IEIs, mostly involving the lympho-reticuloendothelial system, but also other organs, including the CNS [1, 7, 11] (Fig. 17). WAS and AT have the highest incidence of malignancy overall [8, 9], but CNS involvement by malignant processes is more frequent in WAS and XLP1 [11].

This predisposition can result from abnormal DNA repair, increased radiosensitivity, and chromosomal instability, as seen in AT, NBS, Bloom syndrome, DNA ligase IV deficiency, DCLRE1C (Artemis) deficiency, DNA PKcs deficiency, Cernunnos/XLF deficiency, and RIDDLE syndrome [1]. Therefore, exposure to ionizing radiation should be minimized in these patients and even in carriers, both for diagnostic and therapeutic purposes, with MRI being the preferred imaging method [7–9, 11, 17]. When undiagnosed, radiation therapy can have catastrophic results in such patients [11].

In addition to chronic pulmonary disease and progressive neurologic deterioration, lymphoproliferative and solid neoplasms, such as adenocarcinomas, are among the most common causes of death in patients with AT who survive the first decade of life [8, 9, 11]. No increased risk of cancer has been reported in ATLD [7].

Brain tumors have been associated with NBS, particularly medulloblastomas [7, 17], along with an increased risk of lymphoid neoplasms, which occur in more than 40% of patients [11].

The incidence of lymphoproliferative disorders (most commonly non-Hodgkin lymphoma) in WAS is about 2% and 10% at 10 and 30 years of age respectively, with extranodal disease frequently involving the CNS and the lungs [8, 32, 33]. Furthermore, Kaposi sarcoma is seen in 15% of patients with WAS and lymphoproliferative disorders [28, 33].

Other IEIs with increased risk of malignancy, though not necessarily involving the CNS, include selective IgA deficiency and CVID (lymphoma and gastrointestinal neoplasms) [8, 28], cartilage-hair hypoplasia [1, 8], XLA, Shwachman-Diamond syndrome (acute myeloblastic leukemia and myelodysplastic syndrome) [7], CHS (recurrent, aggressive lymphoproliferation with diffuse organ infiltration in the accelerated phase) [11], and AR-HIES (lymphoma, particularly of the CNS, and squamous cell carcinoma (SCC) likely associated with chronic *Human papillomavirus* infection) [8, 14, 25]., Patients with DKC, such as HHS, are at very high risk of leukemia and head and neck or anogenital SCC [47].

### Other features

Given their significant heterogeneity, other neuroimaging findings in IEIs do not neatly fit into the etiopathogenic categories proposed above.

Among them, intracranial hemorrhage occurs in approximately 2% of patients with WAS [32]. It is also seen in cases of HLH [16, 41], particularly during the accelerated phase of CHS [11], and in AR-HIES due to DOCK8 deficiency, especially in the subarachnoid space [7, 10, 16, 25]. Brain microhemorrhages, hemorrhagic strokes, PRES, and intracranial arterial abnormalities, including small-sized aneurysms and transient stenosis associated with eccentric vessel wall thickening and enhancement in the prepontine and basal cisterns, are known neuroimaging features of deficiency of adenosine deaminase 2 (DADA2) [52, 53].

In addition to other cases secondary to infectious or non-infectious vasculitis, predisposition to stroke events has been described in several primary immunodeficiencies. These include SIOD [10, 16], AR-HIES due to DOCK8 deficiency [10, 16], PNP deficiency [7], Blau syndrome [16], DADA2 (mostly lacunar strokes in in the deep grey matter or brainstem) [15, 52–54], LAD2, NOMID/CINCA syndrome [16], Shwachman-Diamond syndrome [14], and AGS type 5 due to SAMHD1 mutations, the latter commonly associated with cerebrovascular disease involving medium and large vessels that result in moyamoya syndrome, stroke and parenchymal hemorrhage [26]. Except for SIOD and AGS, sensorineural hearing loss is a possible manifestation of all of these diseases [7, 16, 52], as well as of Muckle-Wells syndrome and some congenital neutropenias such as elastase deficiency, G6PC3 deficiency and Cohen syndrome [1, 16].

Although not common, ocular disease may occur in some IEIs, sometimes preceding more characteristic disease symptoms [44]. Knowledge of these ocular features can assist in early diagnosis, thereby allowing prompt treatment and improving prognosis. Some notable neuroimaging findings include bilateral optic nerve hypoplasia in Bloom syndrome [44] and optic nerve atrophy observed in NOMID/CINCA syndrome and Roifman syndrome [7, 16].

## Conclusion

IEIs result from specific damaging germline variants that compromise the development and/or function of the immune system. Despite significant advances in genetic diagnosis and treatment, a high degree of clinical suspicion is necessary for diagnosis in many cases, and radiologists still play a critical role in the recognition and monitoring of IEIs.

Neurologic involvement, whether as primary or secondary manifestations, is not uncommon in this heterogeneous group of disorders and should be effectively recognized on imaging studies. This comprehensive review offers a systematic approach to the neuroimaging features of IEIs, emphasizing immune dysregulation through inflammation, neurodegeneration, or malignancy as key phenotypes of several diseases.

Increased awareness and familiarity among clinicians and neuroradiologists are fundamental for timely diagnosis and treatment, ultimately improving patient outcomes and survival.

Integrating large language models with specialized platforms such as the Online Mendelian Inheritance in Man (OMIM) database holds significant potential to assist in differential diagnosis and clinical decision-making, particularly in rare diseases. These models could efficiently analyze clinical and imaging data, cross-referencing it with extensive genetic and medical knowledge to identify possible diagnoses that might otherwise be missed. While such tools represent a promising advancement in personalized medicine by augmenting clinical expertise, final diagnostic decisions must rely on careful human judgment, as artificial intelligence lacks full contextual understanding.

## Data Availability

No datasets were generated or analysed during the current study.

## Abbreviations

AD: autosomal dominant
ADC: apparent diffusion coefficient
ADA: adenosine deaminase
AGS: Aicardi-Goutières syndrome
ANE: acute necrotizing encephalopathy
AR: autosomal recessive
AT: ataxia-telangiectasia
ATLD: ataxia-telangiectasia-like disorder
BTK: Bruton’s tyrosine kinase
CANDLE: chronic atypical neutrophilic dermatosis with lipodystrophy and elevated temperature
CGD: chronic granulomatous disease
CHS: Chédiak-Higashi syndrome
CIDs: combined immunodeficiency disorders
CINCA: chronic infantile neurological cutaneous and articular syndrome
CNS: central nervous system
CRMCC: cerebroretinal microangiopathy with calcifications and cysts
CSF: cerebrospinal fluid
CVID: common variable immunodeficiency disorders
DADA2: deficiency of adenosine deaminase
DKC: dyskeratosis congenita
DNA: deoxyribonucleic acid
DWI: diffusion-weighted imaging
EBV: Epstein-Barr virus
FHLH: familial hemophagocytic lymphohistiocytosis
HHS: Hoyeraal-Hreidarsson syndrome
HIES: hyper-IgE syndromes
HLH: hemophagocytic lymphohistiocytosis
HPS: Hermansky-Pudlak syndrome
RIDDLE: RNF168 deficiency/radiosensitivity, immune deficiency, dysmorphic features, and learning difficulties syndrome
SAP: signaling lymphocyte activation molecule (SLAM) associated protein
sCD25: soluble interleukin-2 receptor alpha-chain
SCID: severe combined immunodeficiency
SIOD: Schimke immunoosseous dysplasia
HSCT: hematopoietic stem cell transplant
HSE: Herpes simplex encephalitis
HSV: *Herpes simplex virus*
ICF: immunodeficiency, centromeric region instability and facial anomalies syndrome
IEI: inborn error of immunity
IFN: interferon
Ig: immunoglobulin
IgRT: immunoglobulin replacement therapy
IKBKG: inhibitor of kappa B kinase gamma
IL: interleukin
IP: incontinentia pigmenti
IUIS: International Union of Immunological Societies
JCV: John Cunningham virus
LAD2: leukocyte adhesion deficiency type 2
MKD: mevalonate kinase deficiency
MRI: magnetic resonance imaging
MTHFD1: methylenetetrahydrofolate dehydrogenase, cyclohydrolase and formyltetrahydrofolate synthetase 1
NADPH: nicotinamide adenine dinucleotide phosphate
NBS: Nijmegen breakage syndrome
NK: natural killer
NOMID: neonatal onset multisystem inflammatory disorder
PHACES: posterior fossa malformations, hemangioma, arterial anomalies, coarctation of the aorta and cardiac defects, eye abnormalities, and sternal defect with or without supraumbilical raphe
PNP: purine nucleoside phosphorylase
PRES: posterior reversible encephalopathy syndrome
SPENCDI: spondyloenchondrodysplasia with immune dysregulation
TLR: Toll-like receptor
TORCH: toxoplasmosis, others (e.g. syphilis, varicella-zoster, parvovirus B19), rubella, cytomegalovirus, and herpes simplex virus
WAS: Wiskott-Aldrich syndrome
XLA: X-linked agammaglobulinemia
XLP1: X-linked lymphoproliferative disease
